# Using Voice Coils to Actuate Modular Soft Robots: Wormbot, an Example

**DOI:** 10.1089/soro.2016.0009

**Published:** 2016-12-01

**Authors:** Markus P. Nemitz, Pavel Mihaylov, Thomas W. Barraclough, Dylan Ross, Adam A. Stokes

**Affiliations:** Stokes Research Group, Institute for Integrated Micro and Nano Systems, School of Engineering, The University of Edinburgh, Edinburgh, United Kingdom.

**Keywords:** modular robotics, voice coil actuator, multidimensional actuator

## Abstract

In this study, we present a modular worm-like robot, which utilizes voice coils as a new paradigm in soft robot actuation. Drive electronics are incorporated into the actuators, providing a significant improvement in self-sufficiency when compared with existing soft robot actuation modes such as pneumatics or hydraulics. The body plan of this robot is inspired by the phylum Annelida and consists of three-dimensional printed voice coil actuators, which are connected by flexible silicone membranes. Each electromagnetic actuator engages with its neighbor to compress or extend the membrane of each segment, and the sequence in which they are actuated results in an earthworm-inspired peristaltic motion. We find that a minimum of three segments is required for locomotion, but due to our modular design, robots of any length can be quickly and easily assembled. In addition to actuation, voice coils provide audio input and output capabilities. We demonstrate transmission of data between segments by high-frequency carrier waves and, using a similar mechanism, we note that the passing of power between coupled coils in neighboring modules—or from an external power source—is also possible. Voice coils are a convenient multifunctional alternative to existing soft robot actuators. Their self-contained nature and ability to communicate with each other are ideal for modular robotics, and the additional functionality of sound input/output and power transfer will become increasingly useful as soft robots begin the transition from early proof-of-concept systems toward fully functional and highly integrated robotic systems.

## Introduction

### Soft robots

The soft robotics community is continuing to make great strides in developing this emerging new class of machines. Many of the reported designs take inspiration from soft-bodied invertebrate animals such as octopi, and in this article, we continue that trend by exploring a new source of inspiration: earthworms. Many of the early developments in soft robotics have resulted in systems, which move slowly, and with a few recent and very notable exceptions,^1–4^ they often require pneumatic or electrical tethers to a fixed location (e.g., a source of compressed air^5^ or combustible mixture of gasses^6^). We previously reported^3^ that to explore unstable or hazardous environments, soft robots must possess the following characteristics: (i) be capable of locomotion; (ii) be capable of movement on unstable terrain such as sand; (iii) be sufficiently inexpensive that they can be abandoned if damaged or contaminated; and (iv) be equipped with sensors and communications systems.

In this article, we report on the use of voice coil actuators for soft robots, and we demonstrate a fully modular system, which meets three of these four criteria. Our system is designed to be completely self-sufficient and with the capacity for locomotion, sensing, communication, and wireless power transfer.

### Actuation mechanisms for soft robots

Mechanical compliance is an inherent property of soft materials and one which confers many advantages when these materials are used in place of those intrinsically more rigid materials (metals, carbon fiber) that are conventionally used for building robots. The use of intrinsically soft materials for structural and actuating components in robots, however, has posed unique challenges. Previously reported soft actuators include electroactive polymers,^7^ shape memory alloys,^8^ and biosynthetic actuators.^9^ Some challenges are that electroactive polymer actuators require a high-voltage source; shape memory alloy actuators are usually slow; and while biosynthetic actuators have progressed far in the last decade, they still require specialized biological processing techniques, such as tissue culture. In this study, we present a composite system, which uses a combination of intrinsic (material) and extrinsic (shape) properties, and which results in a system that is more compliant axially than radially.

### Voice coil actuators for soft robotics

We have developed a modular soft robot with a voice coil electromagnetic actuation system, which uses the same fundamental principles as a hi-fi loudspeaker. The sequential expansion and contraction of a linear chain of segments—each containing an electromagnetic actuator arranged as a voice coil—collectively propel the robot body over a surface. These voice coils consist of a permanent magnet and a moving coil actuator. Interestingly, these actuators are multifunctional; in addition to their primary function at low actuation frequencies, they can also be used across a wide frequency range for (i) sound input/output when configured as a microphone or speaker; (ii) inductive charging; and (iii) intramodule communication (module-to-module using on–off keying [OOK]). By embedding light sensors into the body of Wormbot, we demonstrate another key ability of the earthworm—exteroceptive sensing. Using the onboard light-dependent resistor (LDR) could allow us to explore phototactic behaviors, and we recognize that voice coils could be used as sensors for sound or position and for audio or proprioceptive sensing.

### Recent developments in soft, worm, and modular robotic systems

Worm-like robots have been investigated for at least 30 years.^10–13^ Recent work, which explores similar themes to our Wormbot, includes the work by Wright *et al.* on modular snake robots^14^ and that by Boxerbaum *et al.* on peristaltic motion.^15–17^ Other relevant projects include the work by Lin *et al.* on soft-bodied rolling robots,^18^ exploration of multigait and hybrid soft robots by Shepherd *et al.*,^5^ and work on soft robotic fish by Marchese *et al.*^2^

Wright *et al.*^14^ introduced a new modular snake robot, which used a modified servo actuator, and by attaching a soft polymer skin to their snake robot, the robot was capable of climbing both inside and outside pipes.

Boxerbaum *et al.*^15,16^ reported a soft robot, which used continuous wave peristaltic motion: a locomotive gait found in earthworms. They disproved the assumption that peristaltic motion requires strong anisotropic ground friction and showed that the transition timing between the aerial and ground phases of each individual body part played a crucial role in the amount of slippage and the final robot speed; we use this technique here in our Wormbot.

Shepherd *et al*.^5^ introduced a soft robot comprising entirely soft materials, which was inspired by invertebrates—such as squid, starfish, and worms—that do not have hard internal skeletons.

Marchese *et al.*^2^ introduced a soft-bodied swimming robot, which is capable of continuum-body motion, and by using embedded actuators, their autonomous system was a highly self-sufficient robot.

### Bioinspiration and choice of actuator for Wormbot

Our soft modular robot is inspired by annelids, a subspecies of which is the earthworm. Earthworms comprise a series of segments filled with an incompressible coelomic fluid and each segment is supported by adjacent muscular walls.^19^ In the earthworm, each segment is separated from the next, thus allowing independent actuation. Earthworms possess a set of muscles, which allow individual segments to expand and to contract; the sequential expansion and contraction of segments cause waves passing through the worm, causing peristaltic locomotion.^16,20^

**FIG. 1.**
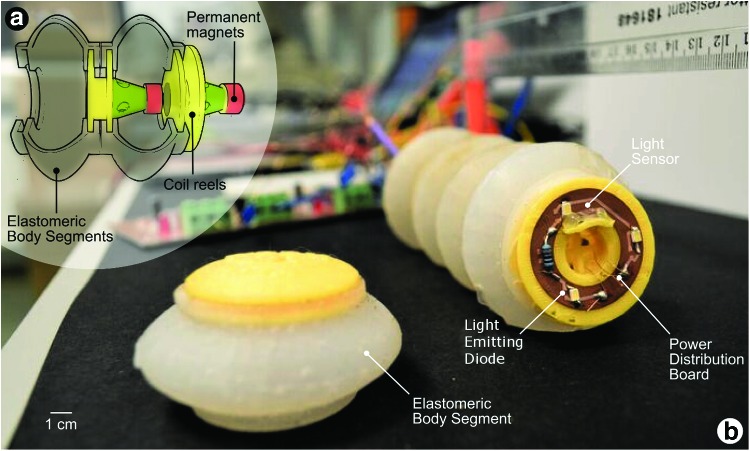
**(a)** A cutaway sketch of one module from Wormbot, showing the connecting elastomeric body segments and a voice coil actuator. **(b)** A photograph showing Wormbot with the rearmost body segment removed to reveal the power distribution board. Color images available online at www.liebertpub.com/soro

In this work, we developed a modular soft robot consisting of elastomeric segments, which are actuated by voice coils: these consist of electromagnetic coils positioned to oppose permanent magnets, as shown in Figure 1. Each segment can be extended or contracted axially, from its rest position, by application of current to the coil; direction of motion is determined by the polarity in which we apply current. Elastomeric body segments connect each module to one another where they provide alignment and give a restoring force. Sequential expansion and contraction of these electromagnetic actuators collectively propel the robot body over a surface.

### Implementing the voice coil actuation system

We wound our own electromagnetic coils on three-dimensional (3D) printed formers and used permanent neodymium magnets in our actuators. These were capable of developing around one Newton of force and could extend and contract fully in the single-digit Hz range. Our early actuator prototypes—consisting solely of electromagnetic coils—required more power than those with permanent magnets, and the increased ohmic heat loss brought the internal temperature above the glass transition temperature of the 3D printed coil former. Each segment in Wormbot contains a coil former, a permanent magnet, and a power distribution printed circuit board to power the coil, as shown in Figure 1b.

### Design of the elastomeric body segments

We developed a thin elastomeric layer, which performs five functions, it (i) contains molded end grooves, which mechanically connect each end of the elastomeric segment to the coil former assembly, as shown in Figure 1; (ii) provides frictional resistance with the surface; (iii) applies a restoring force to return the relative position of segments to the start point; (iv) seals and protects the embedded circuitry; and (v) enables the user to easily replace or change the length of the robot in a modular manner.

### Design of the electronic systems

#### First-generation tethered electronics

**FIG. 4.**
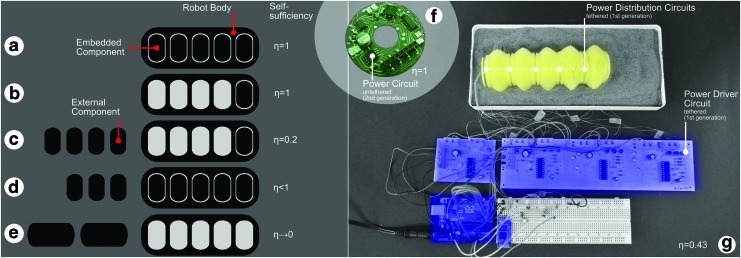
A figure of merit (η) for self-sufficiency can be determined for any robot and serves as a metric for evaluating how integrated the system is, **(a**–**e)** show pictorial examples of how we calculate η. **(a)** For η = 1, all subsystems are fully embedded within the robot and take up all available space. **(b)** Again η = 1 as the available volume inside the robot is greater than that of the embedded components. **(c)** For η = 0.2, some components are not embedded into the robot despite there being enough space. **(d)** For η < 1, all available space in the robot is used, resulting in some required subsystems being tethered. **(e)** For η→0, the subsystems are too large to fit within the robot. **(f)** Our second-generation drive circuit board (PCB) contains a microcontroller, a voltage regulator, and coil drive circuitry. This PCB and small lithium polymer battery fit entirely within one segment, so η = 1. **(g)** Our first-generation power driver circuit and microcontroller sat outside the robot body, while the power distribution circuits were embedded within. Explicit calculations are provided in the Supplementary Data, and we calculate the degree of self-sufficiency as η = 0.43. PCB, printed circuit board. Color images available online at www.liebertpub.com/soro

We divided the first-generation power circuit into a power distribution board and power driver circuit. We integrated the power distribution circuit into each module and attached the external power driver circuit to the soft robot by an electrical tether, as shown in Figure 4g. For this initial tethered robot, we threaded wires through consecutive segments as the robot was assembled, where they then exited through the rear of the robot. Current for the coils was supplied by L6234 drivers arranged as H bridges and incorporating flyback diodes. Three states were possible for each coil: extension, contraction, or an undriven rest state. We used an Arduino Uno microcontroller to supply the logic inputs to the H bridges. We connected a smartphone to the Arduino by Bluetooth and this allowed us to control or reprogram the robot for different actuation patterns. We also equipped our soft modular robot with light-dependent resistors in each power distribution board.

#### Second-generation untethered electronics

We designed a fully integrated printed circuit board (PCB), which contains a microcontroller, voltage regulator, power distribution, and power driver circuit on one board. Each segment of the robot is driven by its own PCB, which can be driven by a small lithium polymer battery, thus demonstrating a fully self-sufficient robot with distributed power and control.

### Embedding components—modular design for self-sufficiency

We define self-sufficiency as measure of the integration of required subsystem components:

\begin{align*}{V_{Robot , components,embedded}}:  = The \ volume \ of \ embedded \ \\ robot \ components\end{align*}

\begin{align*}
{V_{Robot , components,all}}:  = The \ volume \ of \ all \ robot \ components
\end{align*}

\begin{align*}
 { \eta _ { self - sufficiency } } = { \frac { { \int \int \int } \ { f_ { Robot , components,embedded } } \left( { x , y , z } \right) dxdydz }  { { \int \int \int } \ { f_ { Robot , components, all } } \left( { x , y , z } \right) dxdydz } }
\end{align*}

We designed our modular robot with two generations of power circuits. The first generation required the use of external components (shown in Fig. 4g); and the second generation was entirely integrated into the module (shown in Fig. 4f, Supplementary Fig. S7, and Supplementary Video S3). We encountered significantly higher design constraints for the integrated circuit compared with the external circuit, for example, space (height of electronic components); circuit shape (hole in the middle of circuit board); and material compliance (soft reliable circuit material to ensure uninterrupted actuation). We were able to increase our metric for self-sufficiency from η = 0.43 (tethered) to η = 1 (untethered). Using the data from the experiments reported in Supplementary Figures S4, S6 and S8, and using a 300 mAh 7.4 V battery, a fully untethered system is capable of moving 25 body lengths in 12 min before the battery is fully depleted. Full details of these calculations are provided in the Supplementary Data.

## Results and Discussion

### Assembly

We fabricated acrylonitrile–butadiene–styrene plastic molds (for the elastomeric body segments) and coil reels using a 3D printer. We designed the coil reel to provide space for the PCB and with a cone for mounting the permanent magnet, as shown in Figure 1a. We glued the permanent magnet onto the cone using hot glue. We provide details of the coil-winding process in the Experimental section. We used CadSoft Eagle for designing the power distribution boards and we fabricated them on double-sided Cu-FR4-Cu 0.1-mm boards using with an LPKF Protolaser U3 laser micromachining system. We used 3D soft lithography to fabricate the elastomeric body segments using Ecoflex-50 (Smooth-On, Inc.). Figure 1 shows a modular soft robot consisting of five segments. We controlled this robot using custom software on a microcontroller (Arduino Uno).

### Initial testing of Wormbot

The size of the robot when assembled in the relaxed position was 160 mm long and 54 mm in diameter. This size was chosen to provide practical dimensions for manufacture and assembly while ensuring that the magnets and coils remain light enough to be supported by flexible membranes. The resolution of our 3D printer sets the lower size limit for our device, but with improved manufacturing techniques, robots of this type could be made significantly smaller (or indeed much larger), but a full system-level analysis of the complex scaling relationships would be required.

Earthworms have two muscle groups in each segment. An axial group shortens the segment, causing an increase in diameter, while the circumferential group narrows the segment, causing an increase in length. These two opposing groups are required because muscle tissue acts only in one direction (it may pull, but not push). In contrast, electromagnetic actuators may act in both directions: this allowed a design to be developed using only one actuator per segment. The placement of the electromagnetic actuator is analogous to the longitudinal muscle group in an earthworm. Earthworm segments are volume conserving. This causes a well-defined extension as a result of circumferential (or radial) contraction, and vice versa.

Wormbot replicated the motion of an earthworm, but the design of each segment differed from its biological inspiration in two significant ways: (i) a single actuator stands in for two muscle groups and (ii) the body segments are filled with gas, so their volume is not conserved.

### Analysis of locomotion over a variety of surfaces

**FIG. 2.**
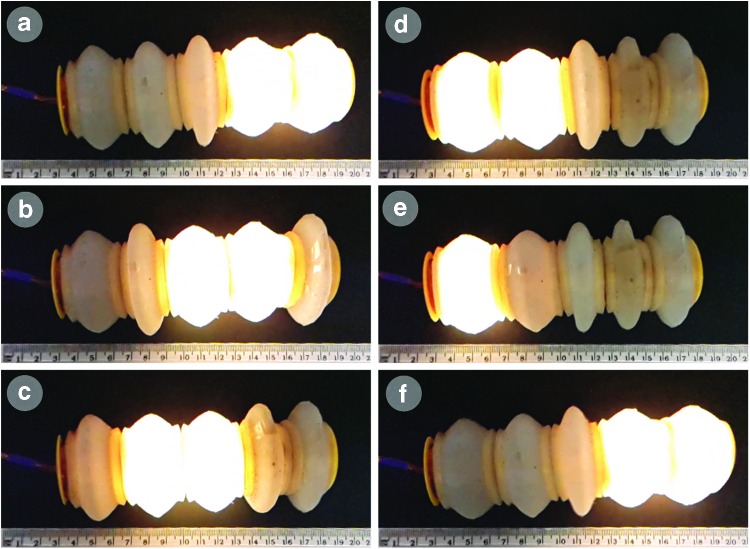
A series of still frames from Supplementary Videos S1 and S2 (Supplementary Data are available online at www.liebertpub.com/soro) showing one cycle of sequential expansion and attraction of modules, the robot moves from *left* **(a)** to *right* **(f)**. These voice coil actuators collectively propel the robot body in relation to the ground using peristaltic locomotion. We embedded indicator light-emitting diodes into the power distribution circuit board so that actuated modules light up when we drive current into the coil. The driving scheme we use is explained in Supplementary Figure S4b. Color images available online at www.liebertpub.com/soro

The speed of the soft robot varied with the sequential expansion and contraction pattern and with the choice of surface material. Figure 2 depicts one cycle of sequential expansion and attraction of modules. We tested the robot with two actuation sequences on five surfaces. In all cases, we found that actuating one segment at a time resulted in the robot traveling further over 10 waves than if we actuated two sequential segments simultaneously. For a sequence of 10-wave (using one segment at a time) actuation, the results show the distances traveled on each surface: polystyrene foam (45 mm); paper (35 mm); aluminum (30 mm); wood (10 mm); and sand (0 mm). Full details are provided in Supplementary Figure S4. We also explored one option for steering this robot using a microservo to drive cables, which run through the body of the robot; Supplementary Figure S9 shows a modular robot equipped with this capability.

### Integrating exteroceptive sensing

We believe that exteroceptive sensing and self-sufficiency are the most important design criteria for designing fully autonomous robot systems that are capable of performing useful tasks. All biological organisms, from bacteria to mammals, sense and communicate locally with one another. Earthworms are capable of measuring the light distribution across their body; they possess sensory cells with a lens-like structure in regions of the epidermis and dermis.^19^

We embedded light sensors into our earthworm-inspired soft modular robot, and our decision was based on a review of sensing in modular robotic systems. Modular robotics has been researched since 1988 and the community has introduced a wide variety of different robot systems. We analyzed the sensing capability of 90 articles, which reported on types of modular robotic systems, and we classified the sensing capability of each system into three classes: no sensing; proprioceptive sensing; and exteroceptive sensing. Supplementary Figure S3 shows the results of our analysis. We discovered that the majority of modular robots do not have exteroceptive sensing capabilities and that in recent years there is a clear trend toward the robotics community incorporating proprioceptive and exteroceptive sensing into their new systems.

### Analysis of the multiple functions of voice coils over a wide range of frequencies

**FIG. 3.**
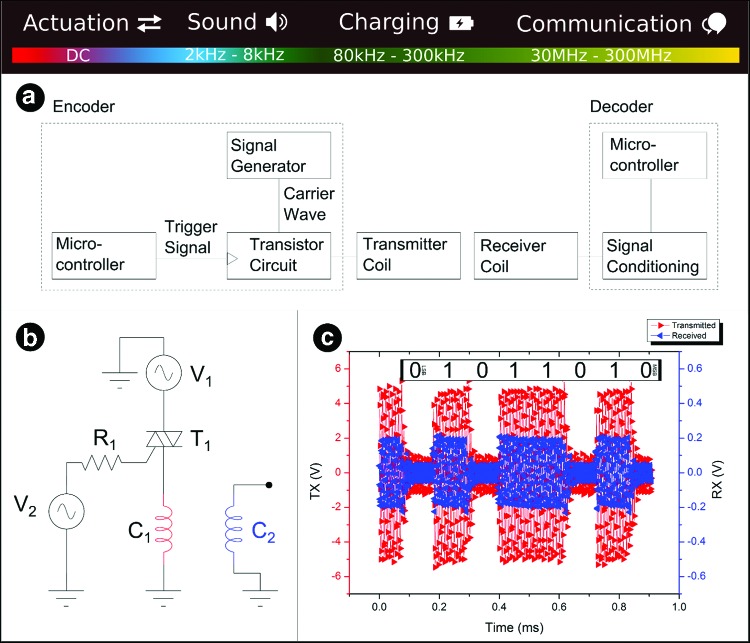
Block diagram, circuit, and results from our intermodule communication experiments. Each coil in the segments of Wormbot can be used for a secondary purpose: as a communication coil. **(a)** We use an on–off keying scheme where the presence of the carrier wave represents a digital one and the absence of the carrier signal represents a digital zero. **(b)** We use a microcontroller to drive a TRIAC transistor, which switches the 50 kHz carrier signal to the transmission coil (C1). If the carrier signal is applied to the transmitter coil, a current flow occurs and the alternating magnetic field causes an induced voltage in the receiver coil (C2). **(c)** A conditioning circuit processes the incoming signal and an ADC input on the microcontroller reads these data in. In this case, we show the transmission from one body segment to the next of the binary ASCII code (01011010) for the character, Z. Color images available online at www.liebertpub.com/soro

The primary function of our voice coils was locomotion, but we found that these could also be used as communication transceivers, audio speakers, microphones, or inductive charging coils. We demonstrated module-to-module communication between consecutive coils in the robot using OOK of a carrier signal. We demonstrated successful communication using 50 kHz carrier signals and a baud rate of 9800 s^−1^. We did not modify the coils for demonstrating communications and they were assembled within the robot throughout the tests. We provided a carrier signal (5 V amplitude) using a signal generator and switched the output using a triac attached to the output pin of a microcontroller, as illustrated in Figure 3b. The transmitted and received waveforms are indicated in Figure 1c. The received signal was attenuated to amplitude of ∼200 mV. If amplified, the received signal could be fed to an integrator, envelope detector, or comparator for signal processing. We used OOK communications to send a binary ASCII character, Z, as a proof of concept for communications. Voice coil actuators are multifunctional devices and although we do not show proof-of-concept experiments, it is clear that these devices could be used for audio input, output, and for wireless power transfer. The multiple functions of these coils can be subdivided by frequency range, and we show a division of the frequency spectrum at the top of Figure 3.

## Conclusions

Our systems differ from previous pneumatically actuated designs, in that our bioinspired modular soft robot (i) actuates electromagnetically, (ii) possesses multifunctional actuators, (iii) contains exteroceptive sensors, and (iv) can be entirely self-sufficient. Advantages of our modular system over our previous pneumatic designs are that (i) no pressurized gas is required; (ii) Wormbot can easily be extended or repaired, and (iii) the modular design makes a robust and redundant system.

We developed a modular soft robotic system with embedded and multifunctional voice coil actuators. Pneumatic actuators require pressurized gas systems and this reduces the robot's ability for self-sufficiency; the result being that majority of pneumatic soft robots are tethered systems. Electromagnetic coils have numerous advantages compared with existing actuators used in soft robots: (i) they are capable of being entirely integrated into self-sufficient systems; (ii) their functionality is frequency dependent and could enable their use for charging, communication, and sound input/output. To further improve the extrinsic softness of Wormbot, future designs could use a polymer such as Polydimethylsiloxane for the coil former, thereby reducing the hard parts to include only the electrical subsystem.

Low-cost robots that use this actuator may find utility in inspection (e.g., pipe inspection) or in search and rescue scenarios (untethered and self-sufficient soft robots using voice coils for locomotion and as microphones for detecting and communicating with survivors). The complete modularity of our modular soft robot will allow this system to be used in scenarios where the robot could be separated into two or more parts and (as long as each subrobot has at least three segments) each would remain capable of performing the original task or even be capable of reconnecting into a larger system again. One significant limitation is that this robot is incapable of motion on sand, perhaps due to the low weight of this system; future research will be directed at locomotion over and digging within bulk granular materials.

## Experimental

### Fabrication of electromagnetic actuators

We bought 0.224 mm insulated copper wire and 10-mm neodymium disc magnets from RS components. We wound each coil to have 820 turns of 0.224 mm diameter enameled copper wire. The resulting winding had an internal diameter of 15 mm, external diameter of 37 mm, and height of 5 mm. We wound the coils using our custom-made winding machine (shown in Supplementary Fig. S1). This machine was controlled by a microprocessor and utilized stepper motors to provide even and consistent winding onto the 3D printed coil formers. We attached permanent magnets to the 3D printed parts using hot glue and secured the magnets using an additional support by wrapping wire around the magnet and through holes in the supporting structure. We quantified the force between the magnet and coil with an ECII-120-type scale (shown in Supplementary Fig. S5).

### Fabrication of the elastomeric body segments

We used a Wanhao Duplicator 4—a low-cost 3D printer—to fabricate a three-part mold (shown in Supplementary Fig. S2). Using this mold, we cast the flexible body segment walls with EcoFlex-50 (Bentley Advanced Materials). EcoFlex-50 has a tensile strength of 2.2 MPa and stiffness of 82 kPa (measured at strain of 1). We vacuum degassed the EcoFlex, then we poured it into the mold and allowed it to settle under gravity. We then degassed the filled mold before curing at 60°C for 20 min in an oven.

### Fabrication of the control circuitry

We bought double-sided Cu-FR4-Cu 0.1-mm boards from LPKF Laser and Electronics AG and used an LPKF Protolaser U3 UV laser micromachining system to manufacture the circuit boards. The circuit boards were designed with CadSoft Eagle.

### Fabrication of the integrated sensory system

We bought Silonex NSL-19 M51 LDR 2-pin TO-18 light-dependent resistors from RS components and soldered these to the power distribution PCB.

### Experiments to test multifunctional voice coil actuators

We made a wireless serial communications link using an Arduino Uno microcontroller and two signal generators (Thurlby Thandar 1 GHz and Phillips PM5135). We wrote a custom microcontroller script for data transmission, the experimental setup is shown in Figure 3b. The coils represent two successive segments in the Wormbot.

Copies of all CAD, PCB, and software files are provided in the Supplementary Data.

## Supplementary Material

Supplemental data

Supplemental data

Supplemental data

Supplemental data

Supplemental data

Supplemental data

Supplemental data

Supplemental data

Supplemental data

Supplemental data

Supplemental data

Supplemental data

Supplemental data
