# Supplementary figures and images for: Using Voice Coils to Actuate Modular Soft Robots: Wormbot, an Example
Source: Soft Robot. 2016 Dec 1;3(4):198–204. doi: 10.1089/soro.2016.0009 (PMC5180079; doi:10.1089/soro.2016.0009)

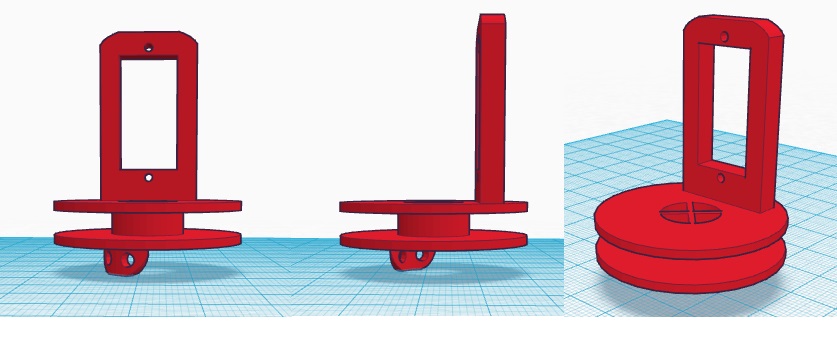

Supplement: Supplemental data [file Supp_Data.zip › Supplemental Information/3D Printer Parts/.picasaoriginals/1.1.jpg]

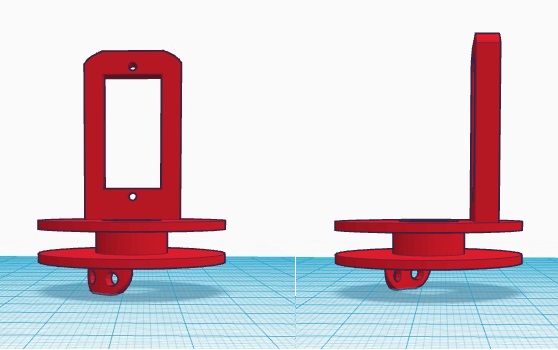

Supplement: Supplemental data [file Supp_Data.zip › Supplemental Information/3D Printer Parts/.picasaoriginals/1.jpg]

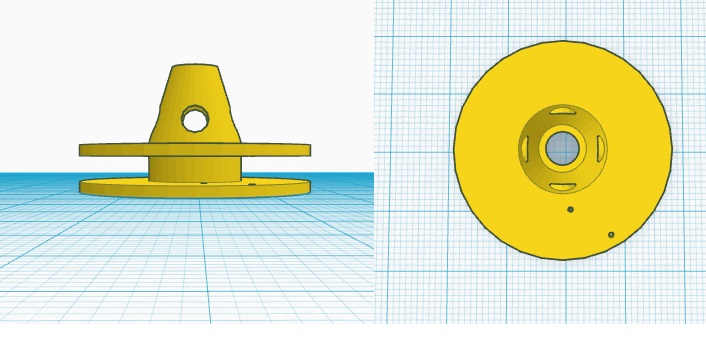

Supplement: Supplemental data [file Supp_Data.zip › Supplemental Information/3D Printer Parts/.picasaoriginals/reel 1.jpg]

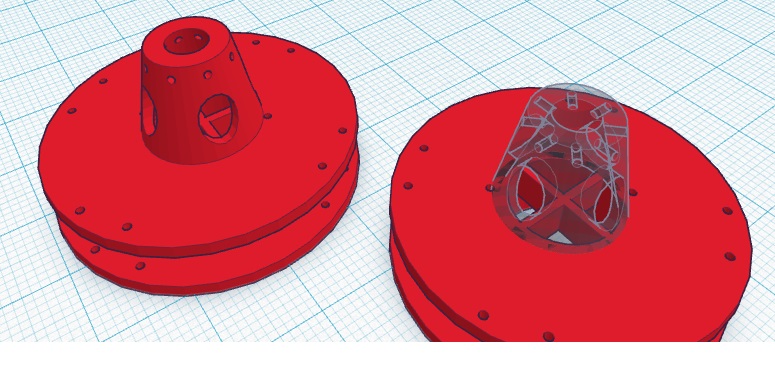

Supplement: Supplemental data [file Supp_Data.zip › Supplemental Information/3D Printer Parts/.picasaoriginals/reel 2.jpg]

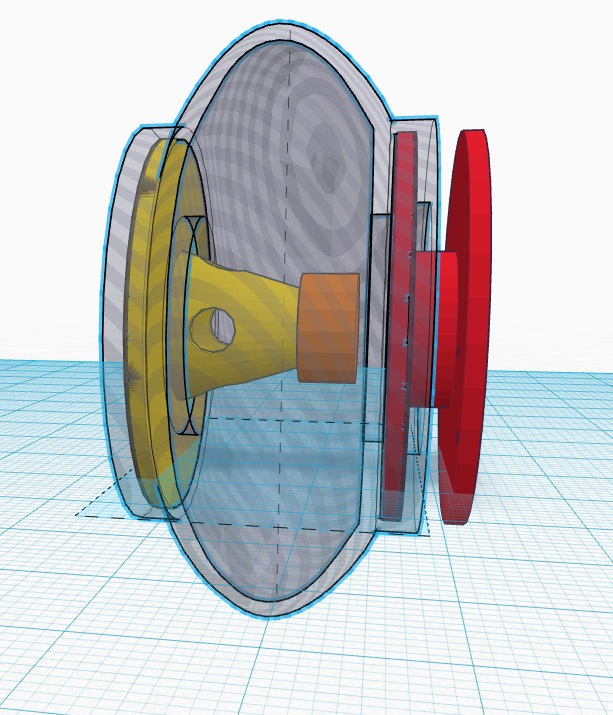

Supplement: Supplemental data [file Supp_Data.zip › Supplemental Information/3D Printer Parts/.picasaoriginals/system 2.jpg]

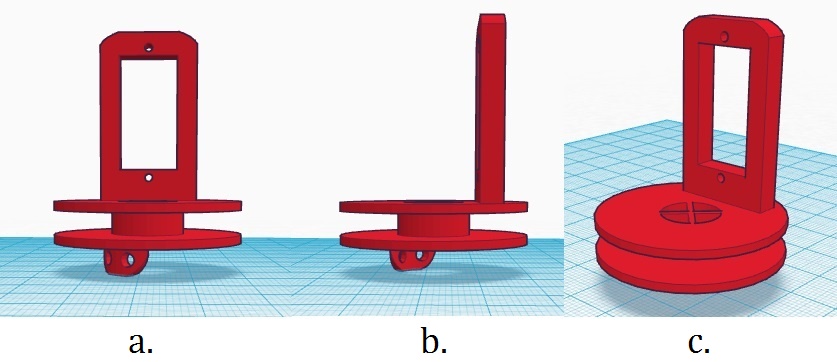

Supplement: Supplemental data [file Supp_Data.zip › Supplemental Information/3D Printer Parts/1.jpg]

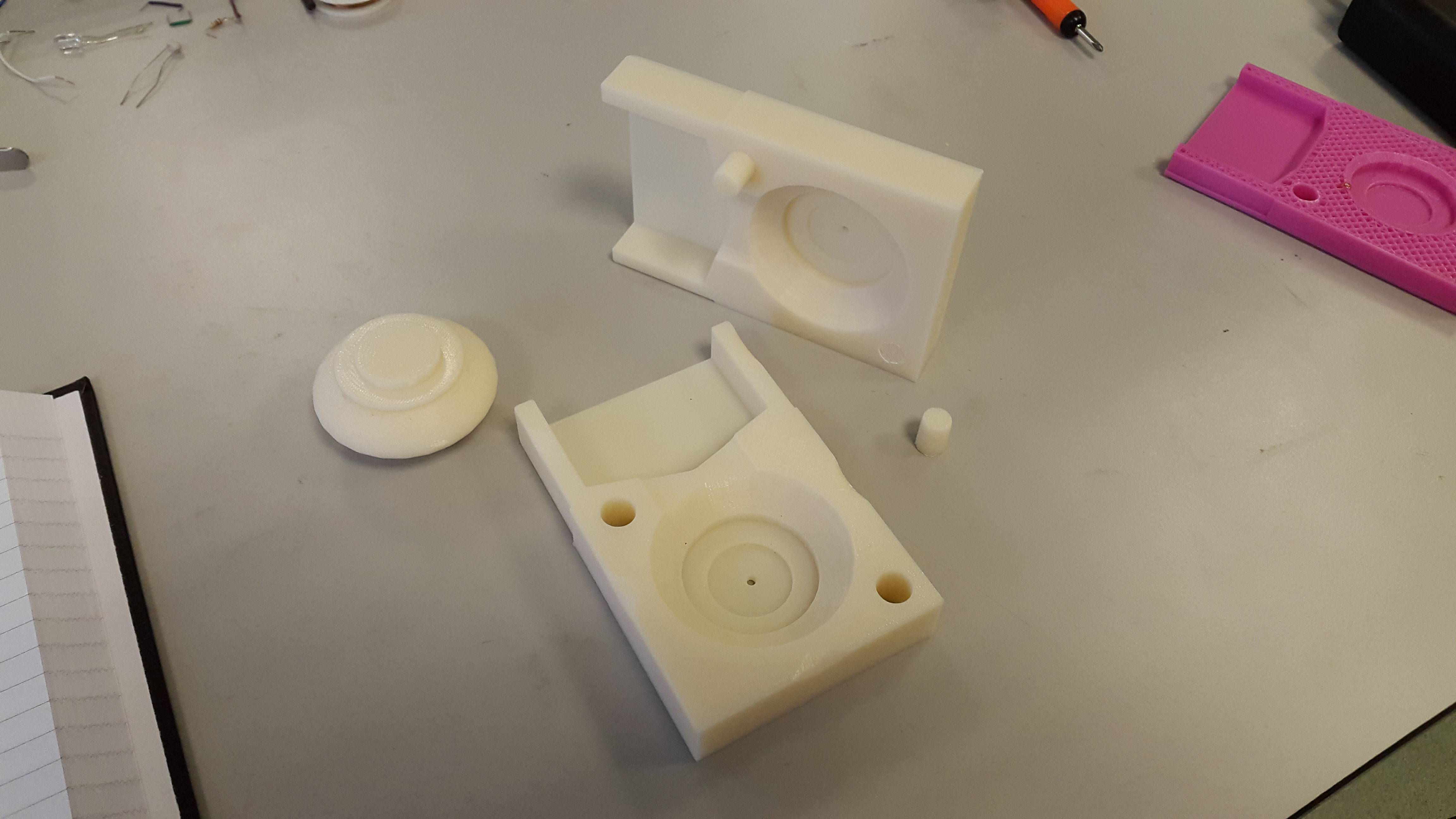

Supplement: Supplemental data [file Supp_Data.zip › Supplemental Information/3D Printer Parts/20141028_121243.jpg]

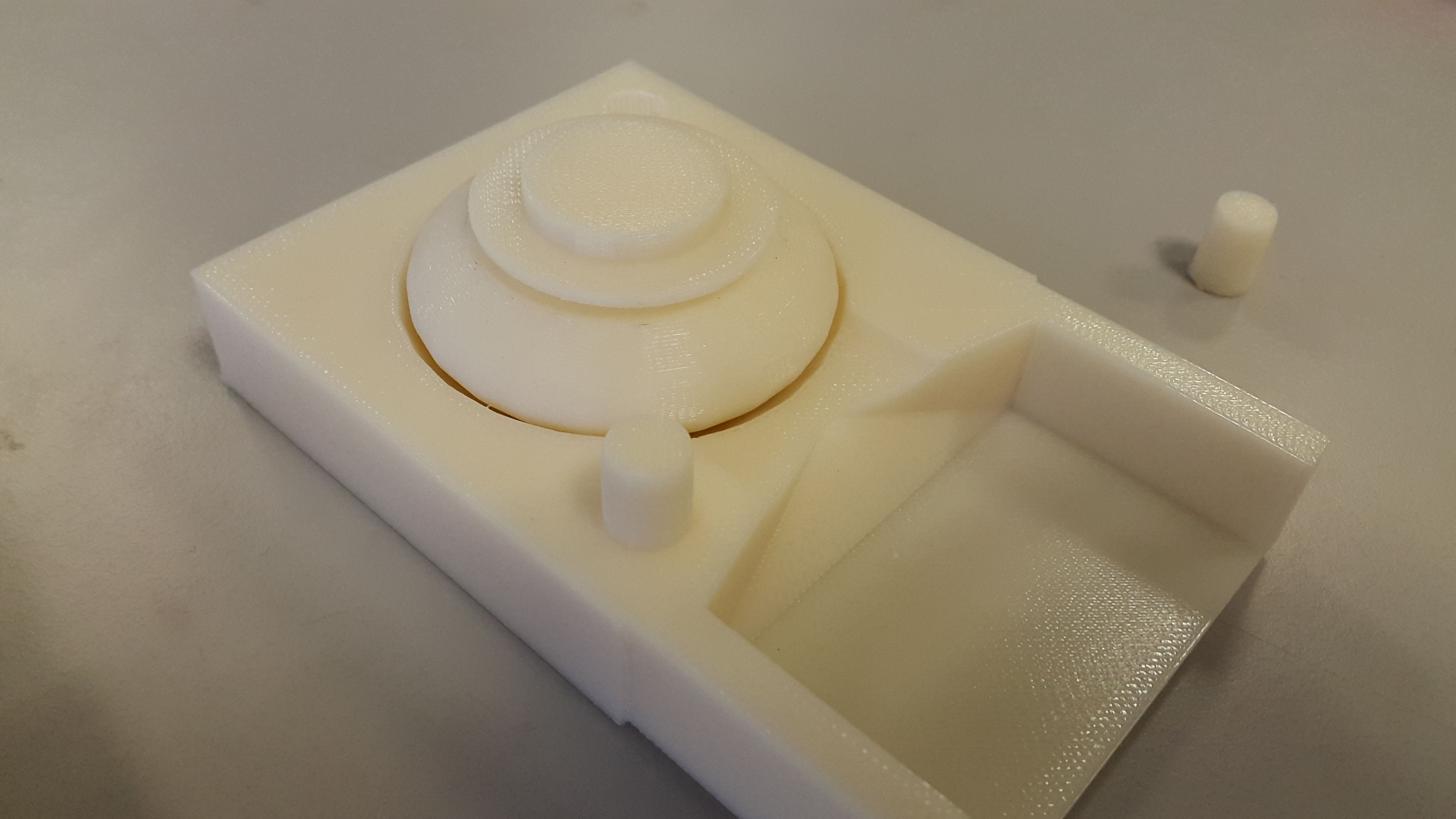

Supplement: Supplemental data [file Supp_Data.zip › Supplemental Information/3D Printer Parts/20141028_121301.jpg]

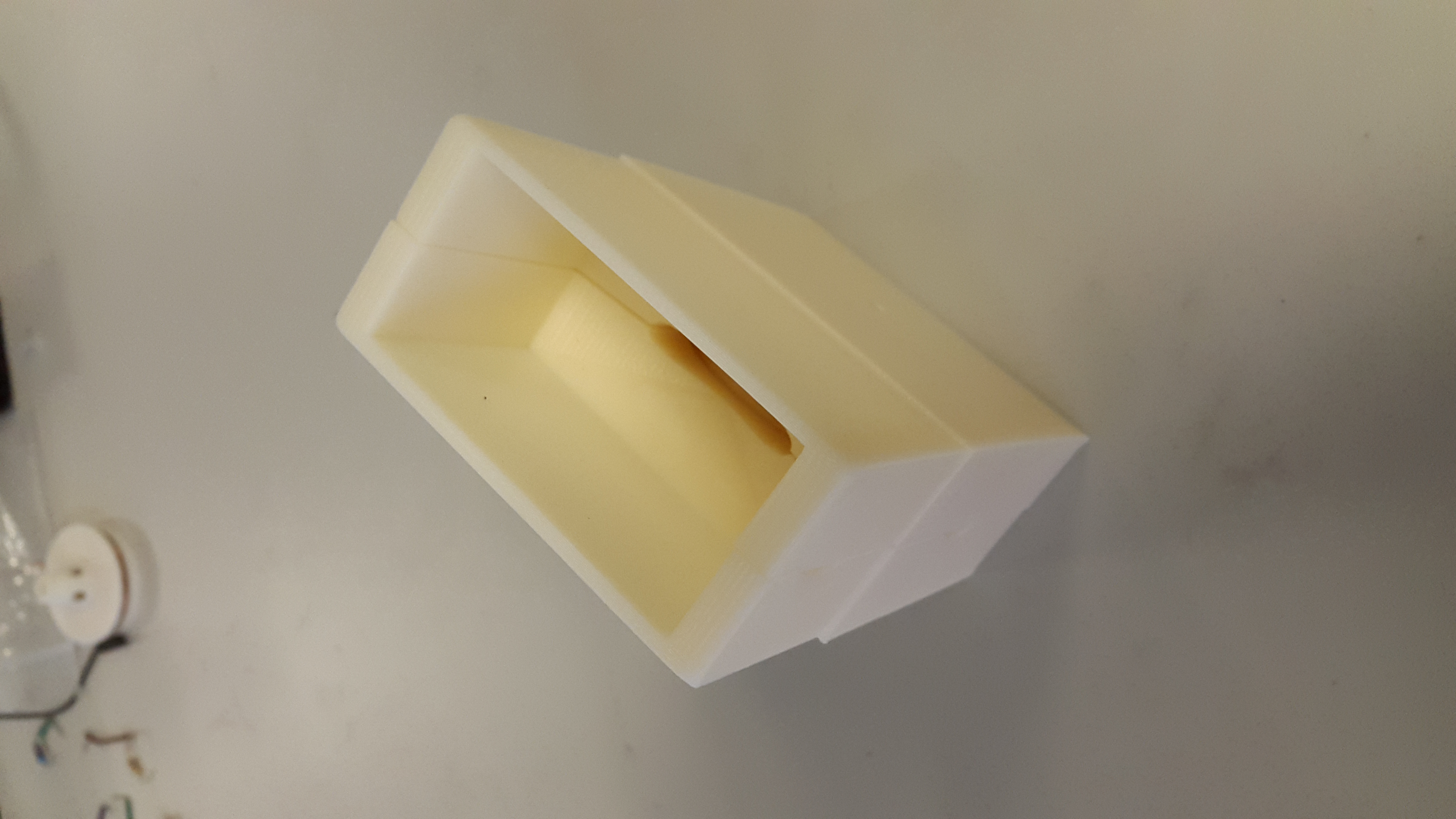

Supplement: Supplemental data [file Supp_Data.zip › Supplemental Information/3D Printer Parts/20141028_121321.jpg]

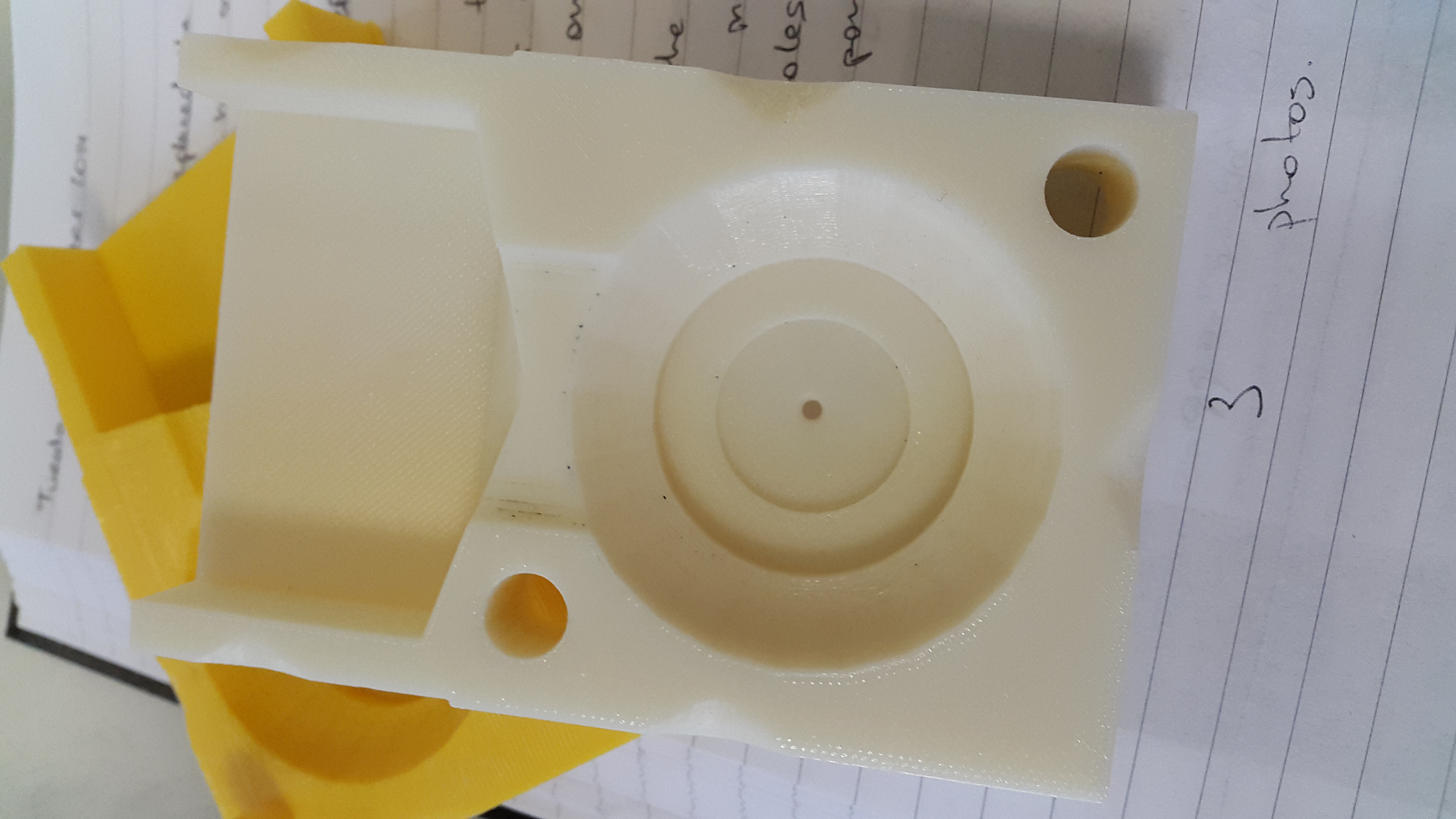

Supplement: Supplemental data [file Supp_Data.zip › Supplemental Information/3D Printer Parts/20141028_121844.jpg]

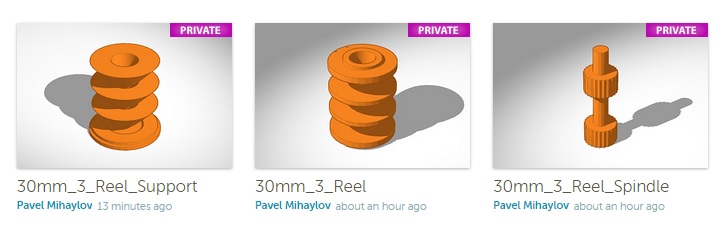

Supplement: Supplemental data [file Supp_Data.zip › Supplemental Information/3D Printer Parts/30mm_Tripple_reel/3pple.jpg]

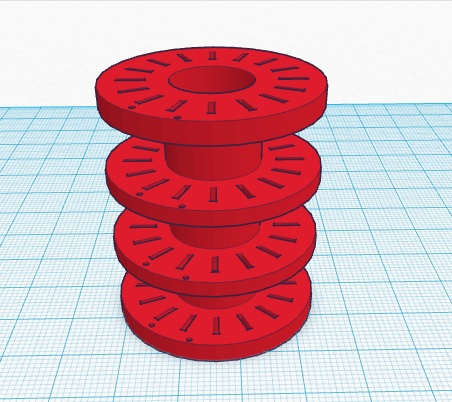

Supplement: Supplemental data [file Supp_Data.zip › Supplemental Information/3D Printer Parts/30mm_Tripple_reel/v2/Untitled.jpg]

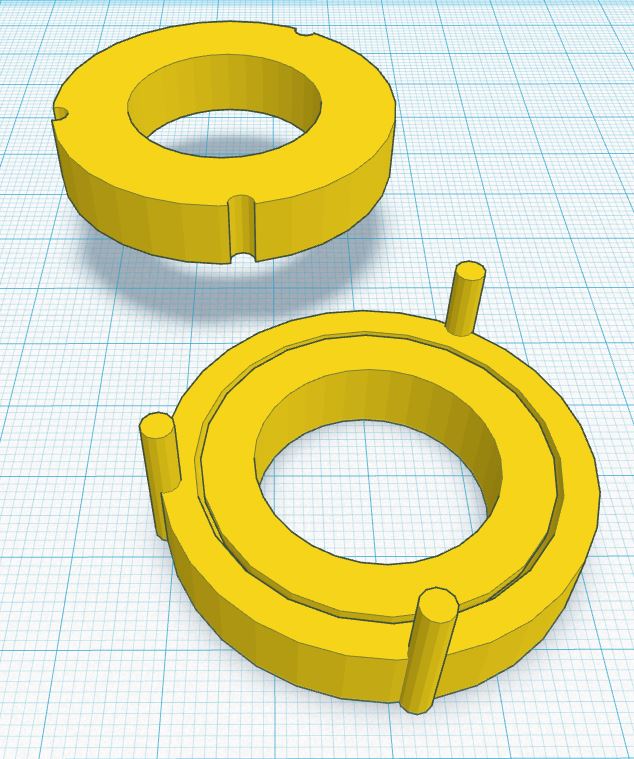

Supplement: Supplemental data [file Supp_Data.zip › Supplemental Information/3D Printer Parts/All moulds/1.JPG]

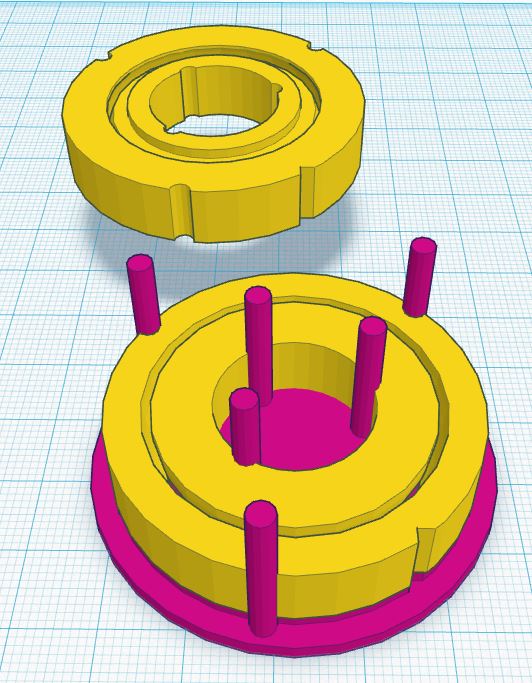

Supplement: Supplemental data [file Supp_Data.zip › Supplemental Information/3D Printer Parts/All moulds/2.JPG]

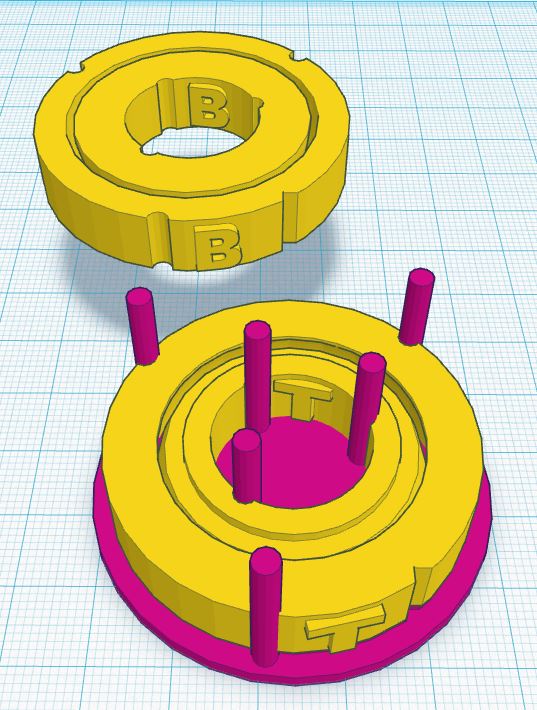

Supplement: Supplemental data [file Supp_Data.zip › Supplemental Information/3D Printer Parts/All moulds/3.JPG]

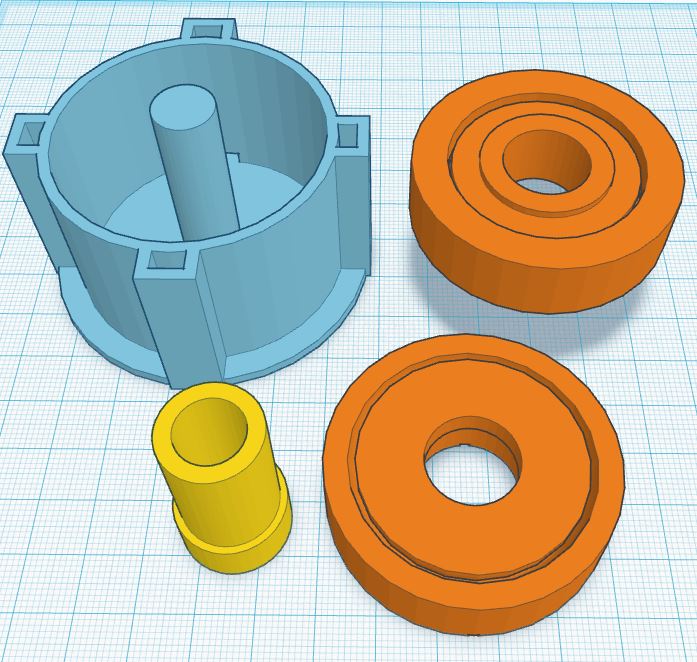

Supplement: Supplemental data [file Supp_Data.zip › Supplemental Information/3D Printer Parts/All moulds/4.JPG]

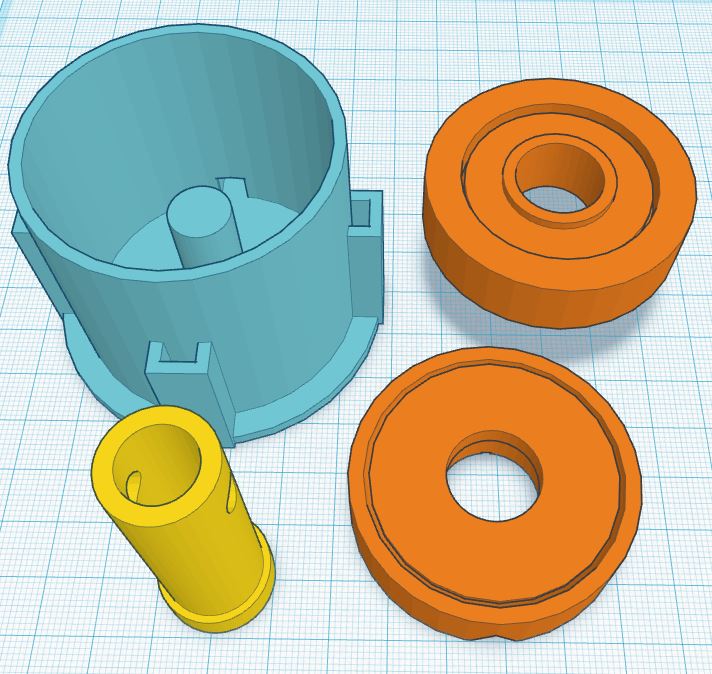

Supplement: Supplemental data [file Supp_Data.zip › Supplemental Information/3D Printer Parts/All moulds/5.JPG]

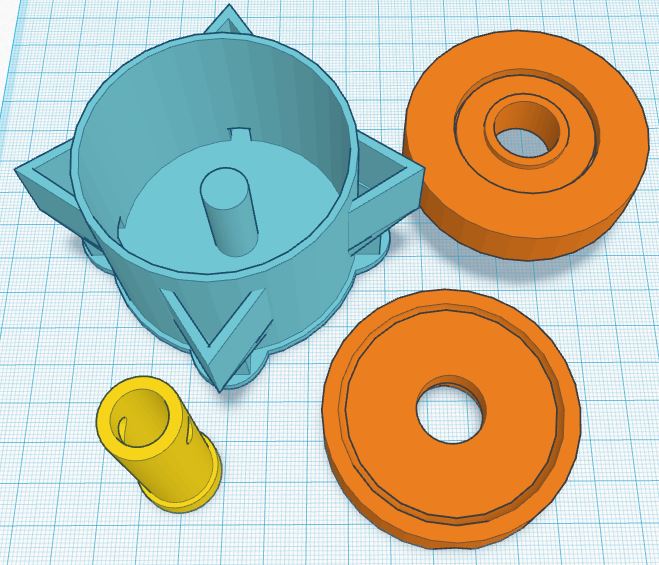

Supplement: Supplemental data [file Supp_Data.zip › Supplemental Information/3D Printer Parts/All moulds/6.JPG]

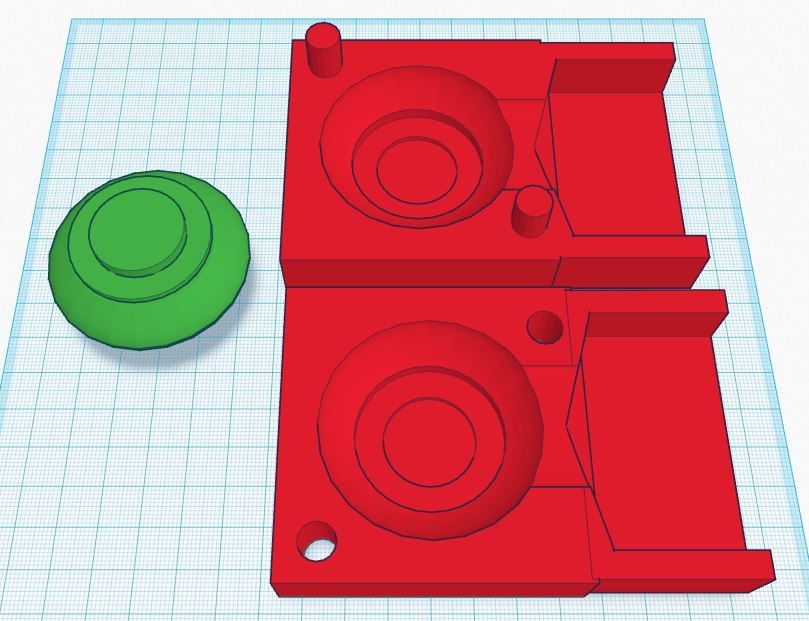

Supplement: Supplemental data [file Supp_Data.zip › Supplemental Information/3D Printer Parts/All moulds/7.JPG]

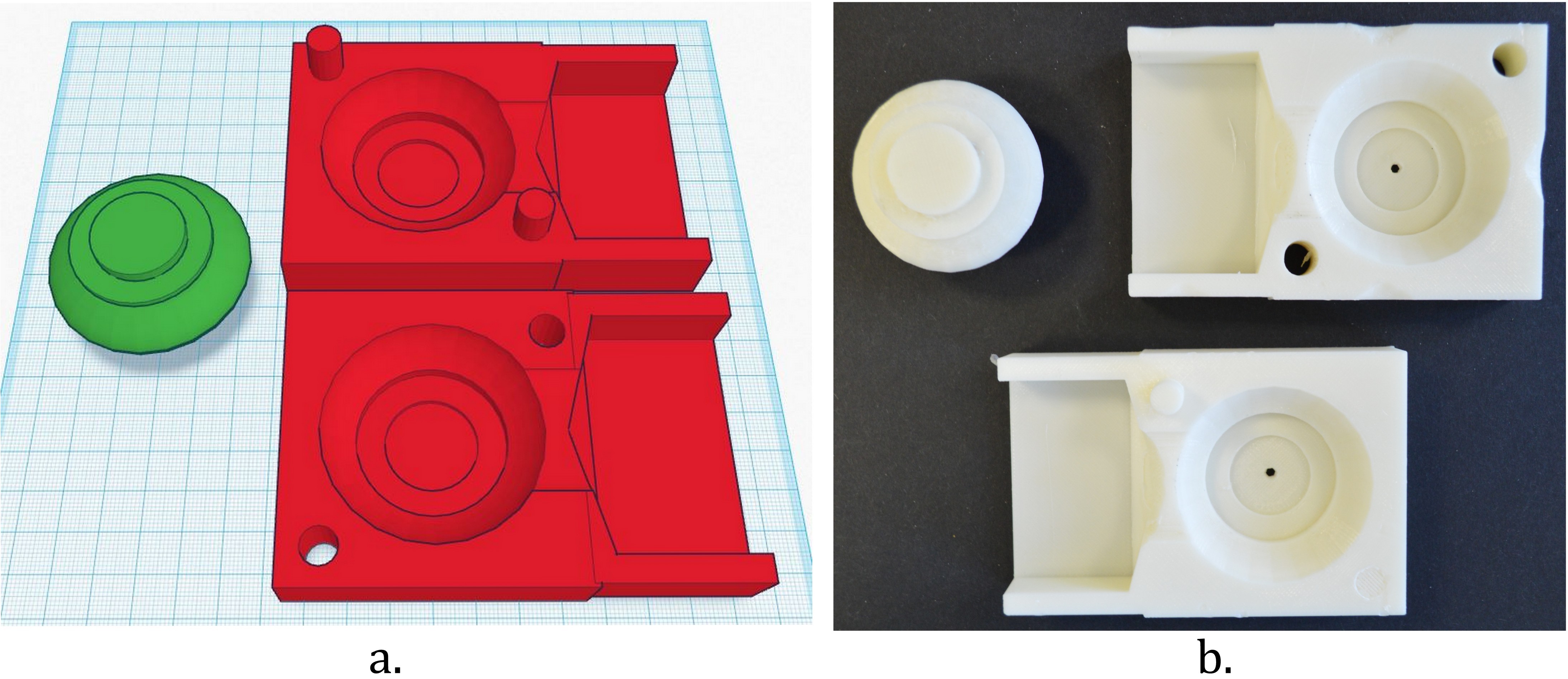

Supplement: Supplemental data [file Supp_Data.zip › Supplemental Information/3D Printer Parts/All moulds/All moulds2.jpg]

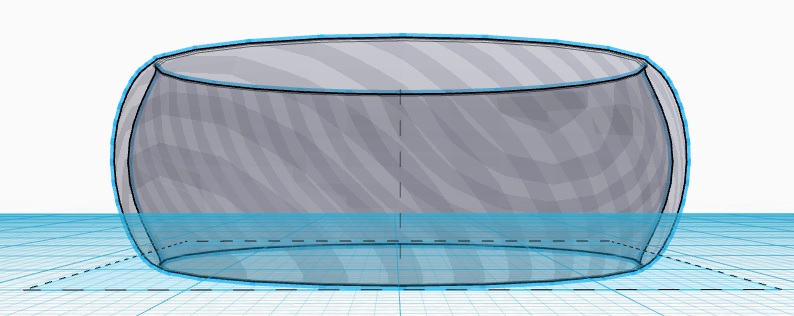

Supplement: Supplemental data [file Supp_Data.zip › Supplemental Information/3D Printer Parts/All moulds/i1.jpg]

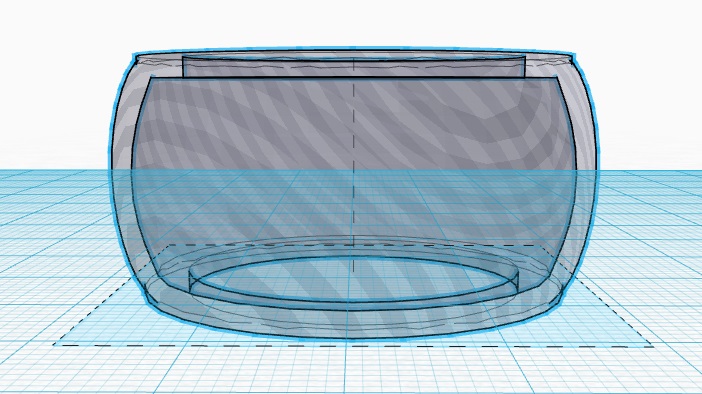

Supplement: Supplemental data [file Supp_Data.zip › Supplemental Information/3D Printer Parts/All moulds/i2.jpg]

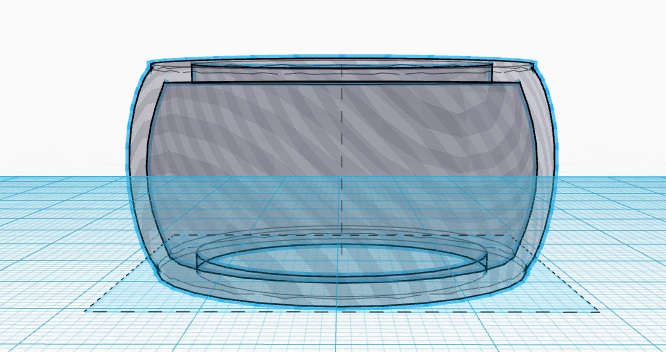

Supplement: Supplemental data [file Supp_Data.zip › Supplemental Information/3D Printer Parts/All moulds/i3.jpg]

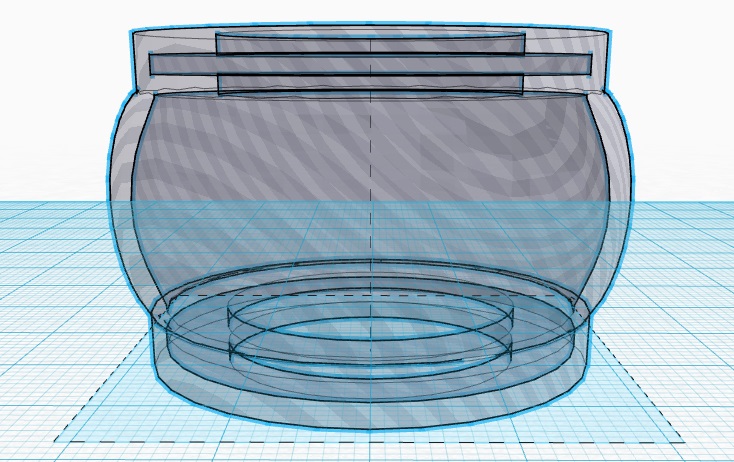

Supplement: Supplemental data [file Supp_Data.zip › Supplemental Information/3D Printer Parts/All moulds/i4.jpg]

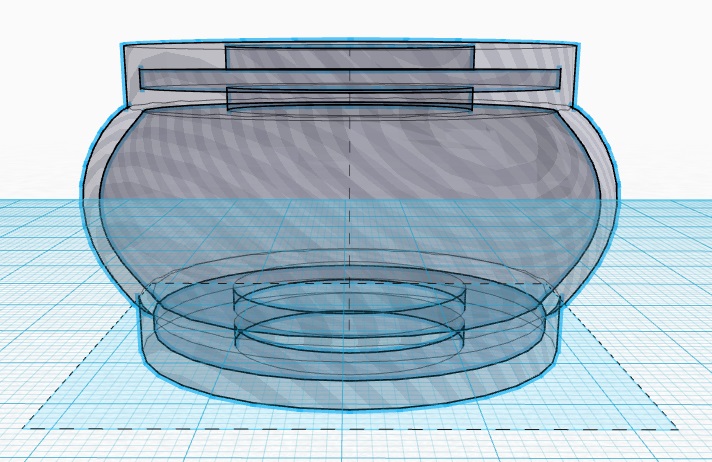

Supplement: Supplemental data [file Supp_Data.zip › Supplemental Information/3D Printer Parts/All moulds/i5.jpg]

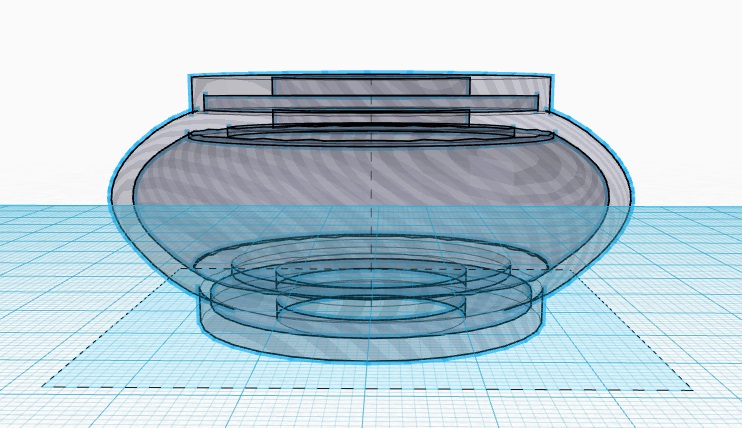

Supplement: Supplemental data [file Supp_Data.zip › Supplemental Information/3D Printer Parts/All moulds/i6.jpg]

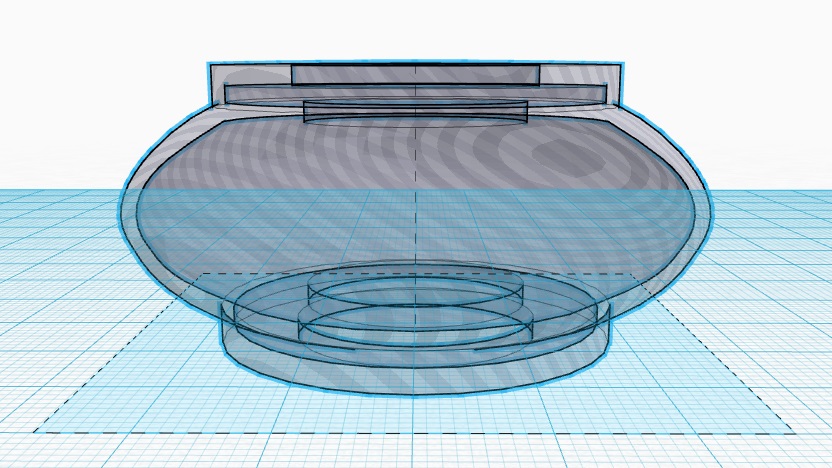

Supplement: Supplemental data [file Supp_Data.zip › Supplemental Information/3D Printer Parts/All moulds/i7.jpg]

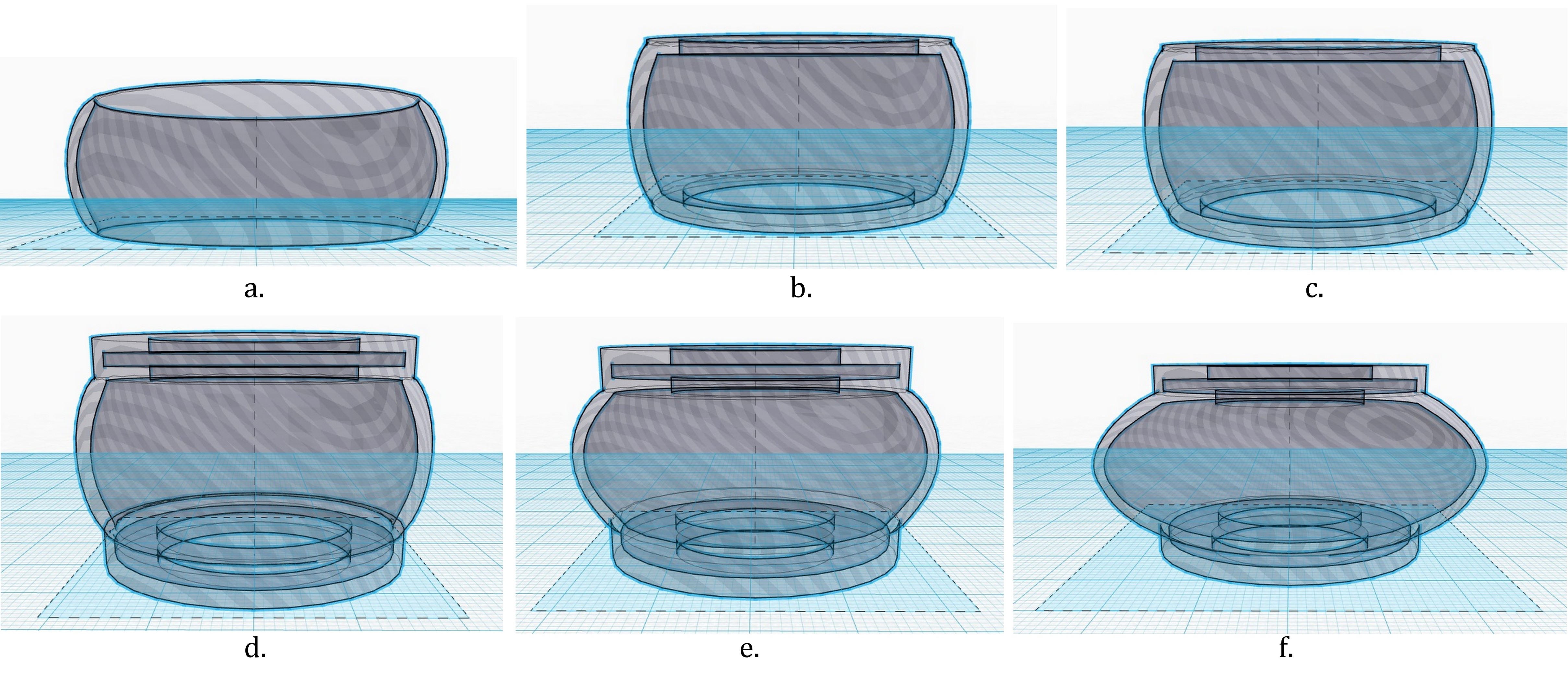

Supplement: Supplemental data [file Supp_Data.zip › Supplemental Information/3D Printer Parts/All moulds/insides.jpg]

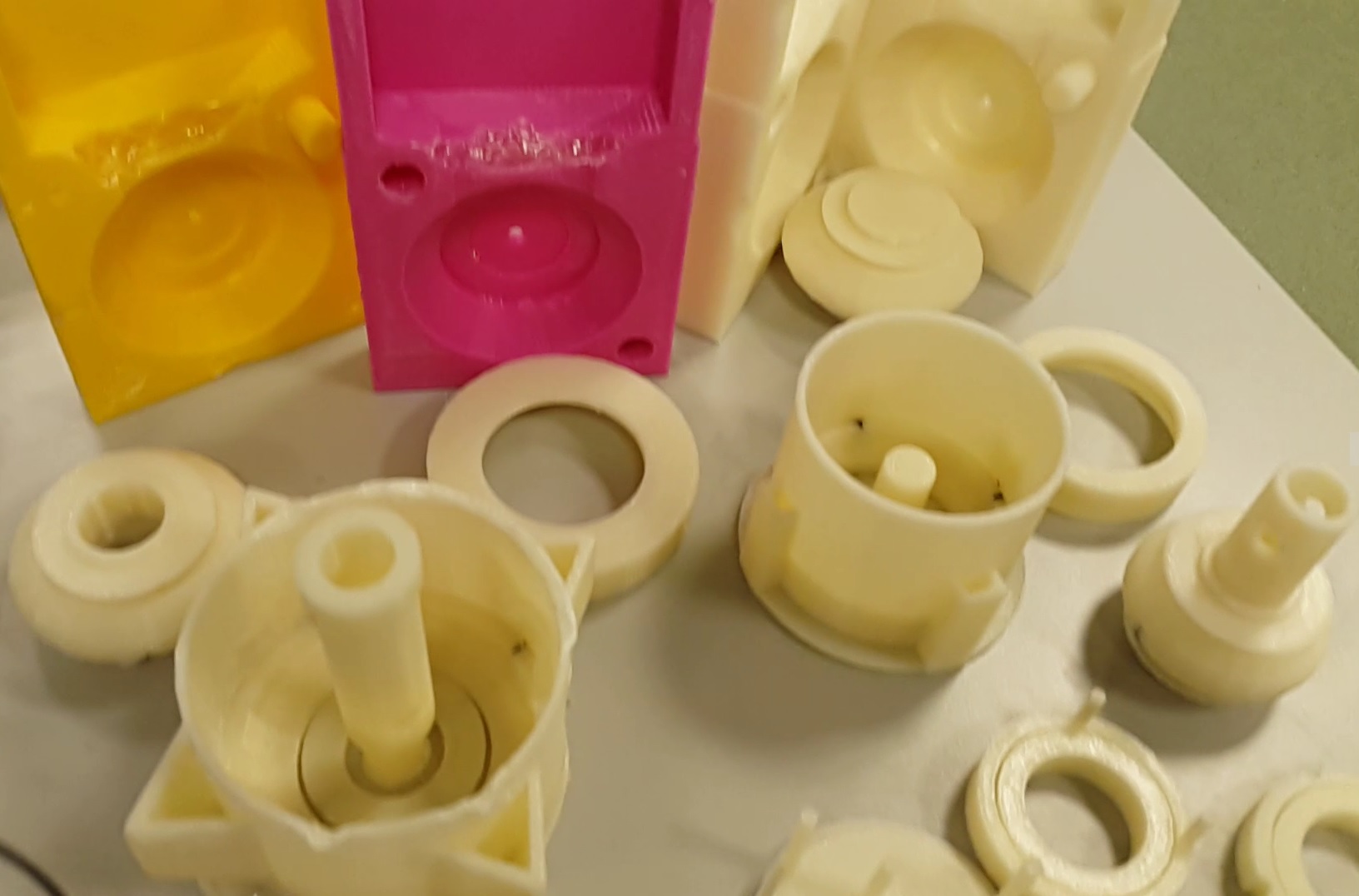

Supplement: Supplemental data [file Supp_Data.zip › Supplemental Information/3D Printer Parts/All moulds/moulds.jpg]

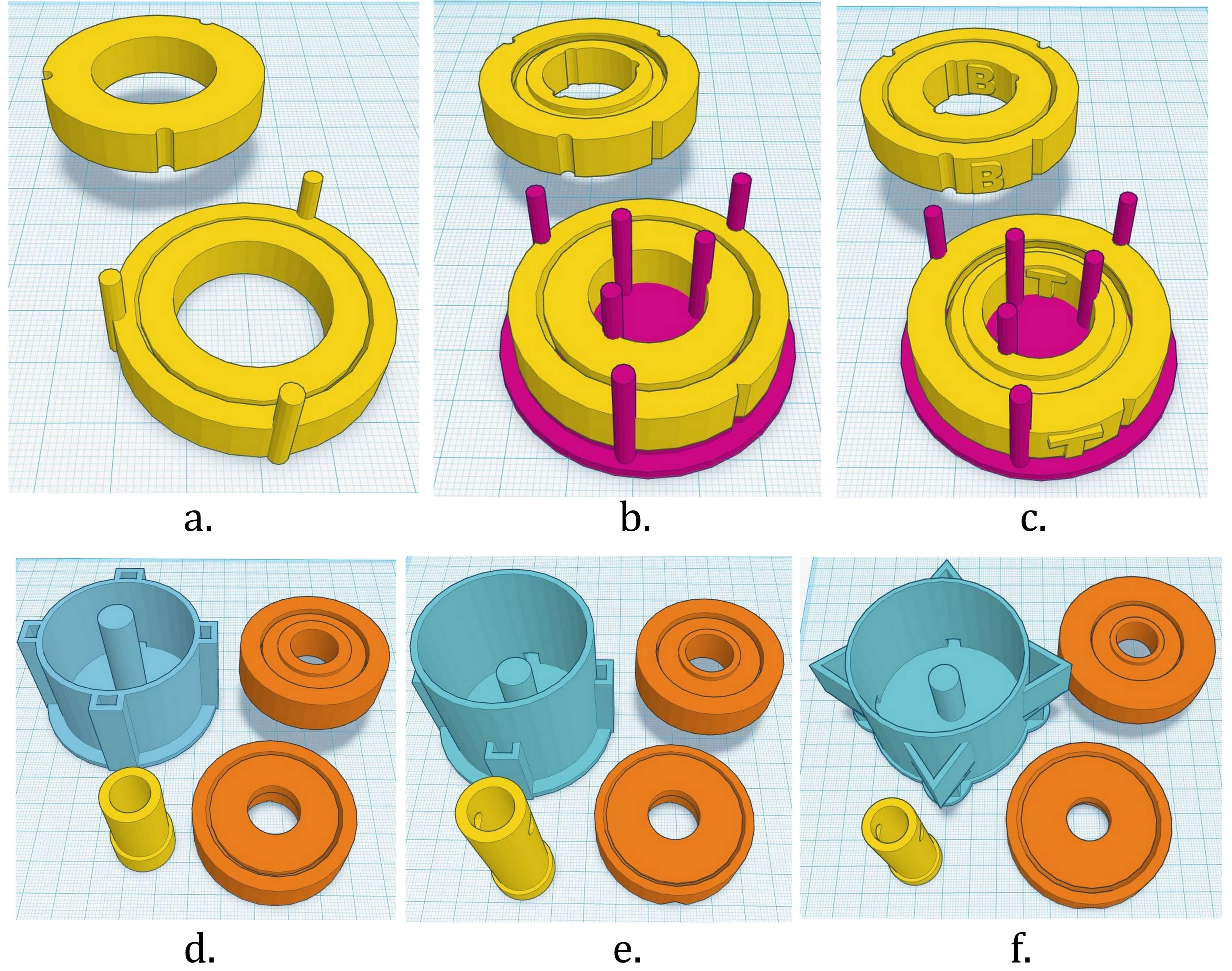

Supplement: Supplemental data [file Supp_Data.zip › Supplemental Information/3D Printer Parts/All moulds/six.jpg]

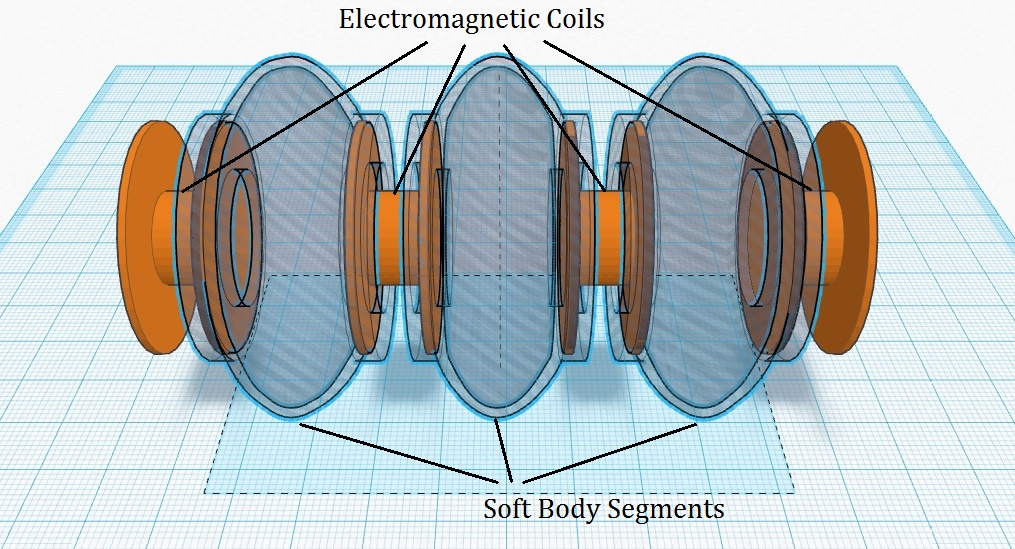

Supplement: Supplemental data [file Supp_Data.zip › Supplemental Information/3D Printer Parts/body wall.jpg]

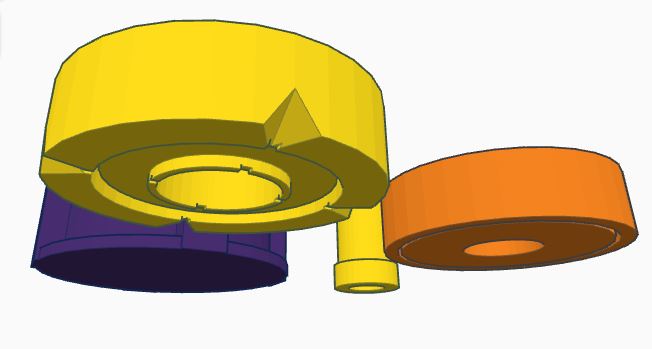

Supplement: Supplemental data [file Supp_Data.zip › Supplemental Information/3D Printer Parts/Capture.JPG]

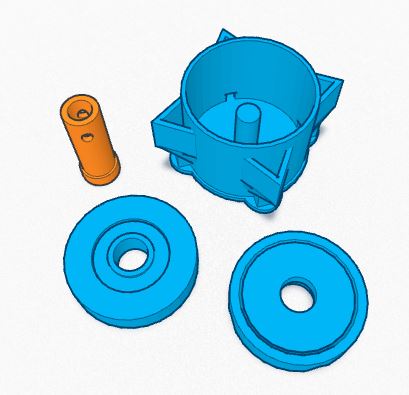

Supplement: Supplemental data [file Supp_Data.zip › Supplemental Information/3D Printer Parts/Capture2.JPG]

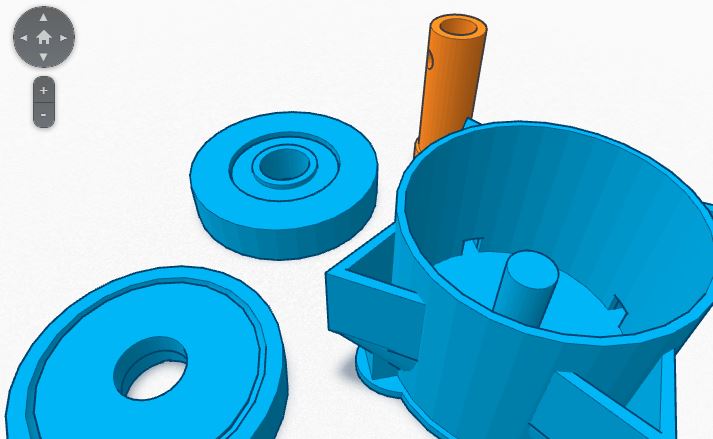

Supplement: Supplemental data [file Supp_Data.zip › Supplemental Information/3D Printer Parts/Capture3.JPG]

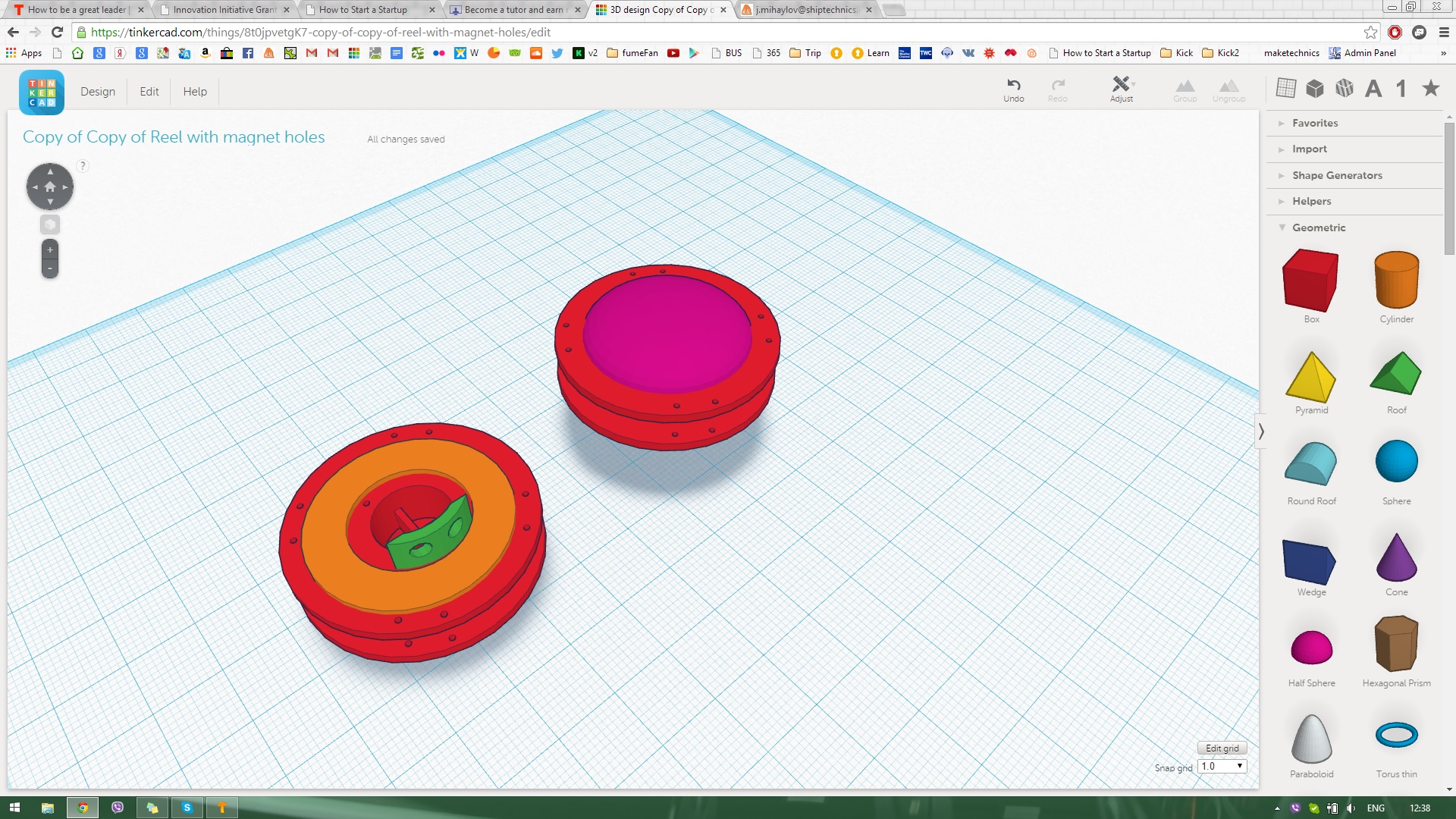

Supplement: Supplemental data [file Supp_Data.zip › Supplemental Information/3D Printer Parts/font_end.jpg]

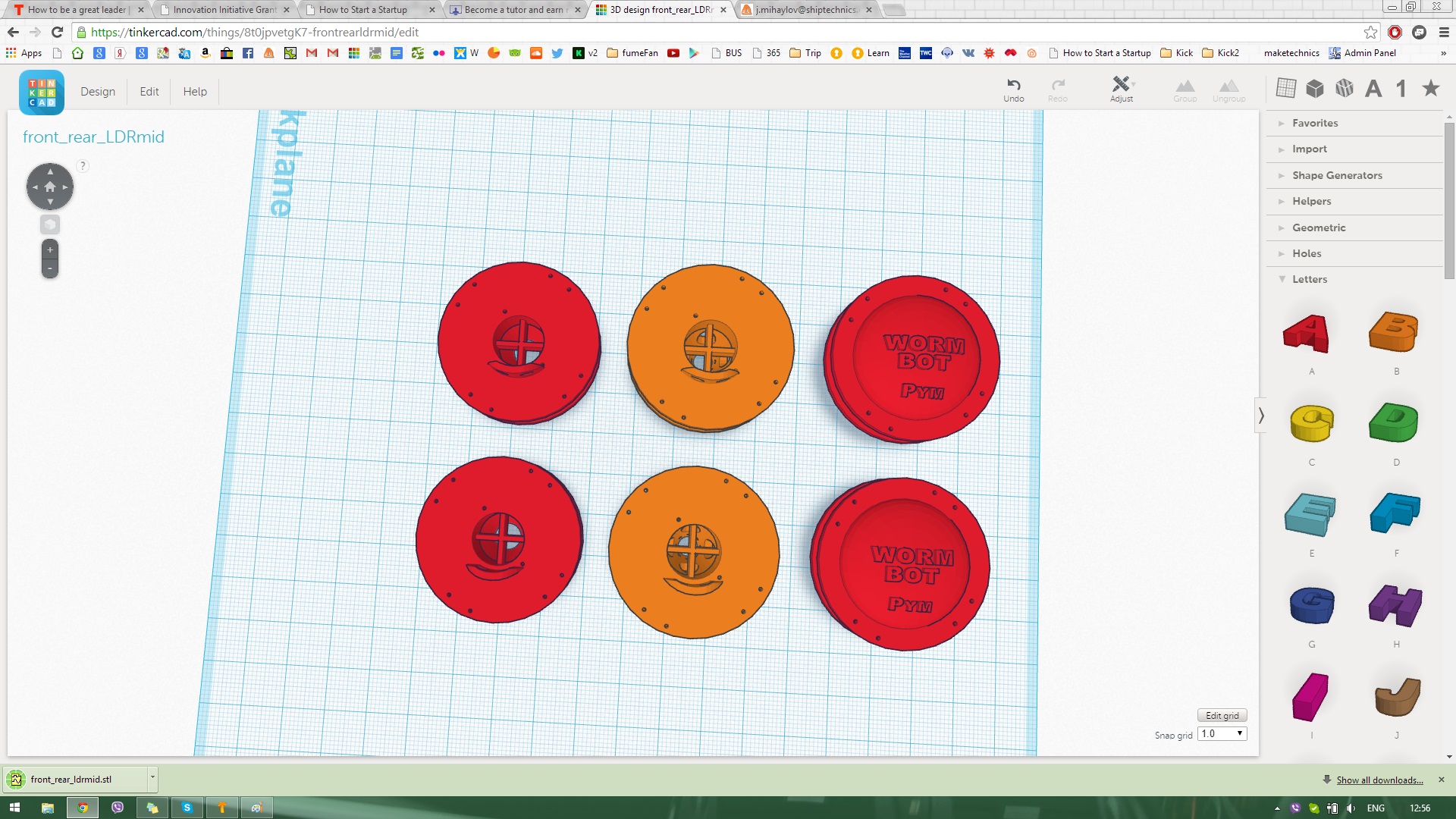

Supplement: Supplemental data [file Supp_Data.zip › Supplemental Information/3D Printer Parts/font_end_LDRmid.jpg]

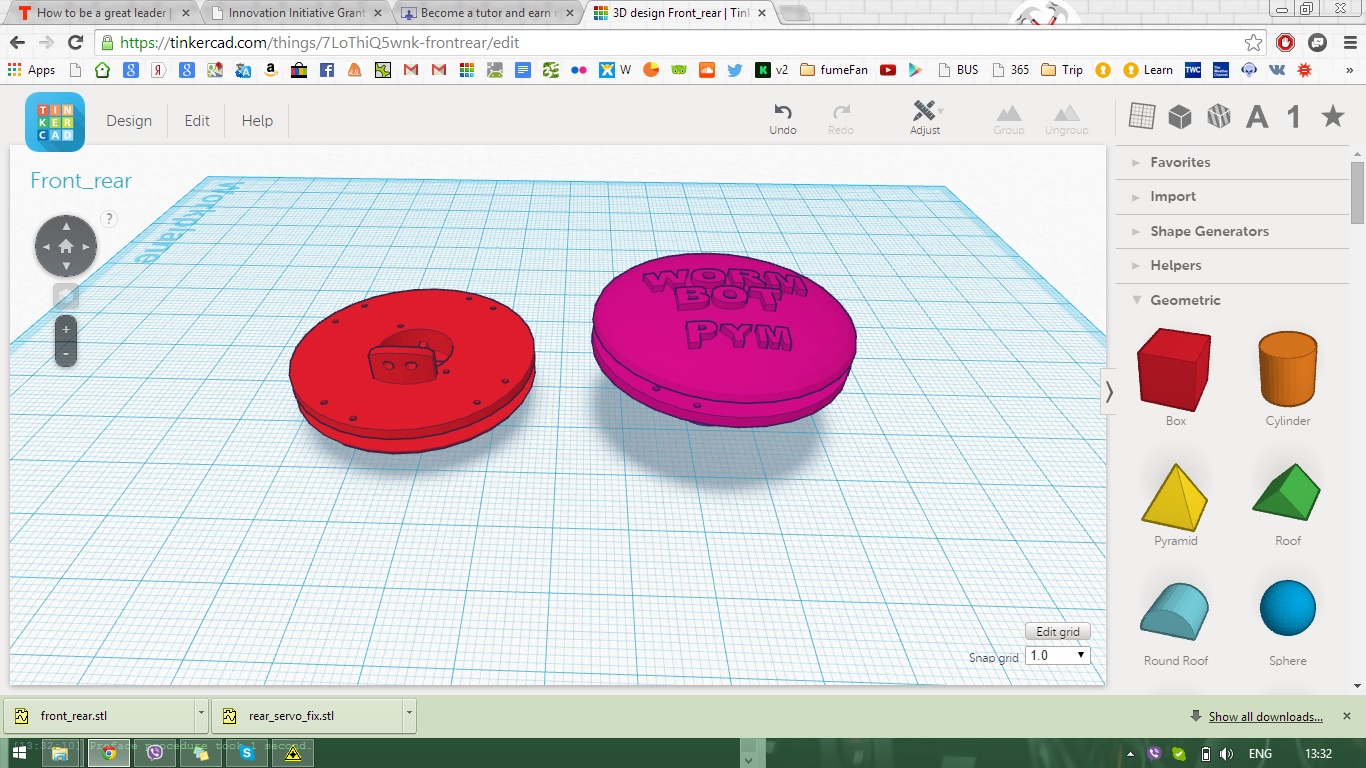

Supplement: Supplemental data [file Supp_Data.zip › Supplemental Information/3D Printer Parts/front_rear.jpg]

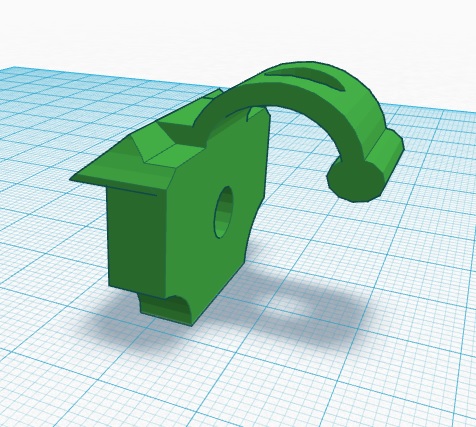

Supplement: Supplemental data [file Supp_Data.zip › Supplemental Information/3D Printer Parts/hook.jpg]

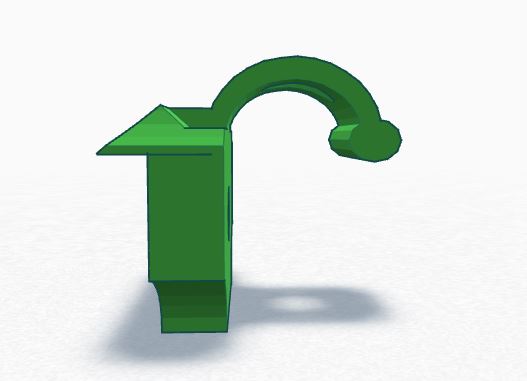

Supplement: Supplemental data [file Supp_Data.zip › Supplemental Information/3D Printer Parts/hook2.jpg]

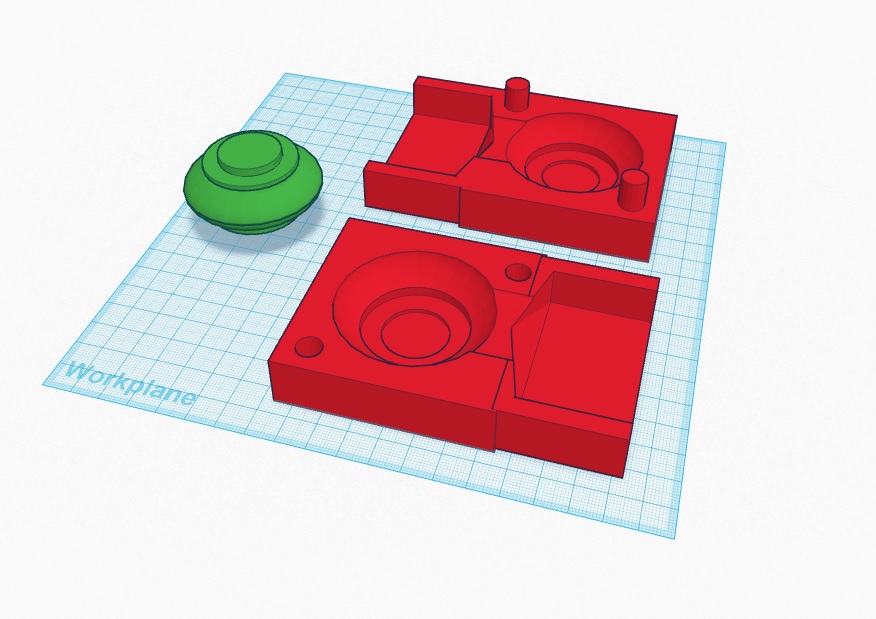

Supplement: Supplemental data [file Supp_Data.zip › Supplemental Information/3D Printer Parts/Mould standing.jpg]

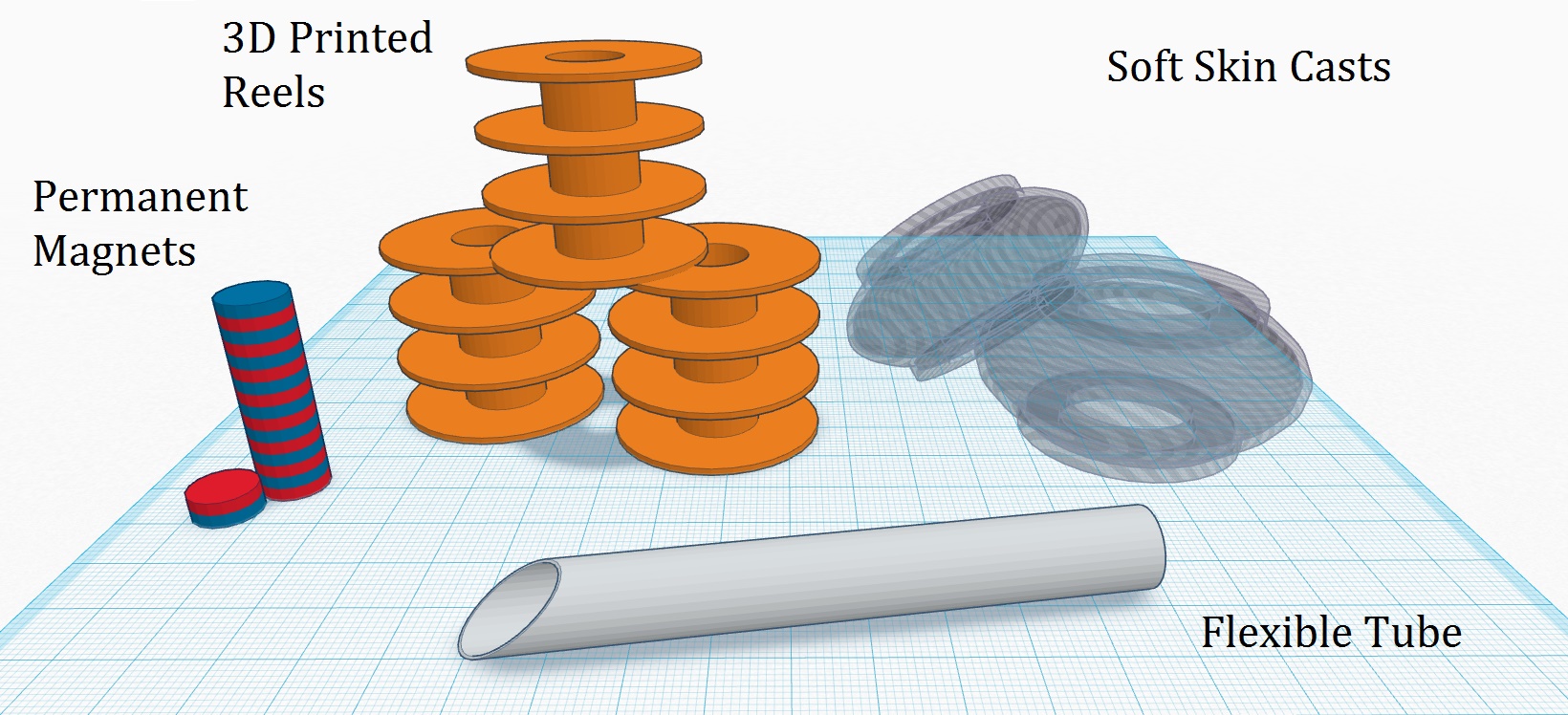

Supplement: Supplemental data [file Supp_Data.zip › Supplemental Information/3D Printer Parts/prop sys 3 parts.jpg]

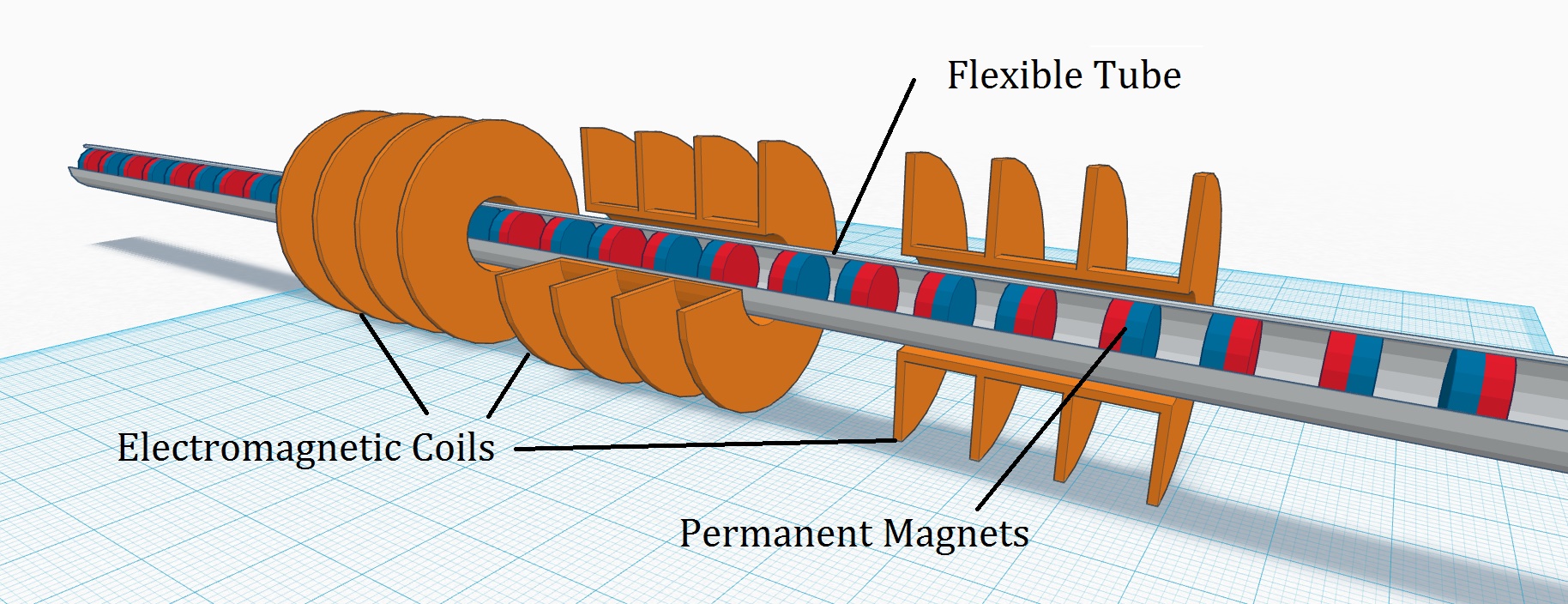

Supplement: Supplemental data [file Supp_Data.zip › Supplemental Information/3D Printer Parts/prop sys 3.jpg]

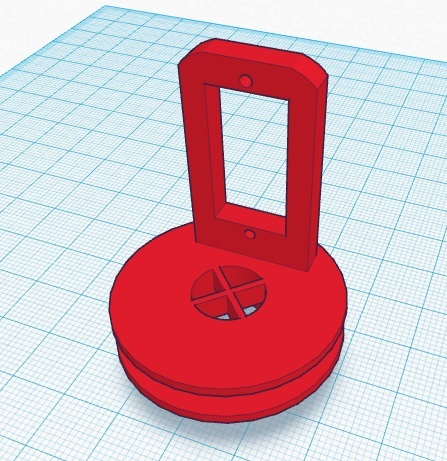

Supplement: Supplemental data [file Supp_Data.zip › Supplemental Information/3D Printer Parts/rear_servo_fix.jpg]

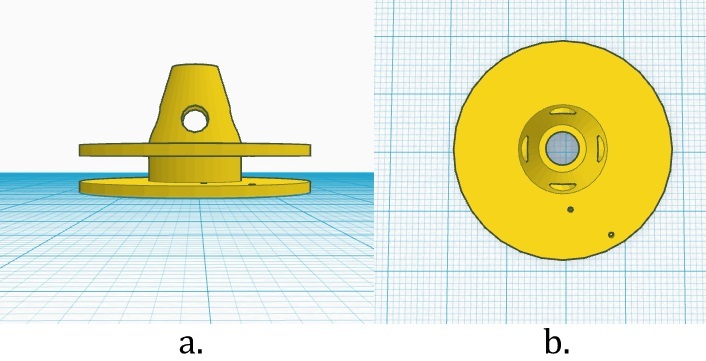

Supplement: Supplemental data [file Supp_Data.zip › Supplemental Information/3D Printer Parts/reel 1.jpg]

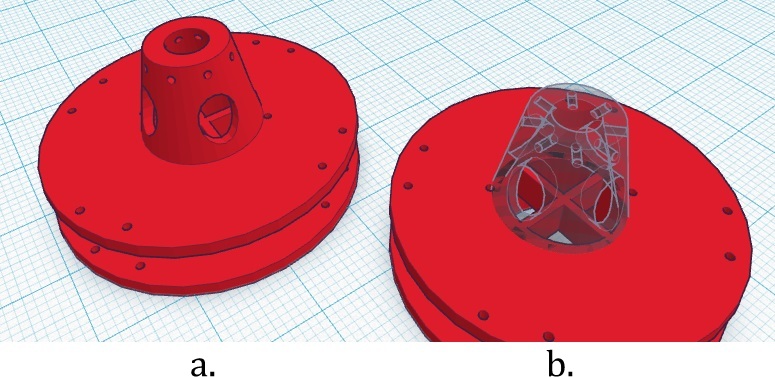

Supplement: Supplemental data [file Supp_Data.zip › Supplemental Information/3D Printer Parts/reel 2.jpg]

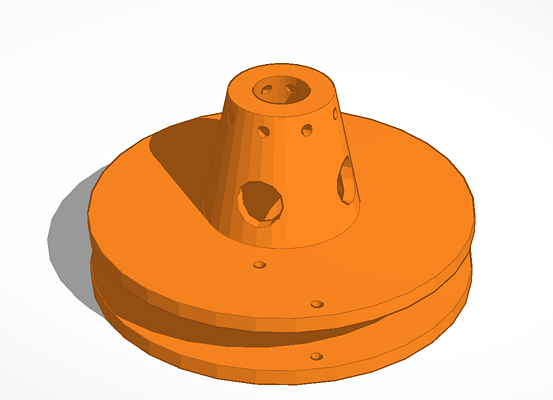

Supplement: Supplemental data [file Supp_Data.zip › Supplemental Information/3D Printer Parts/reel with holes.bmp]

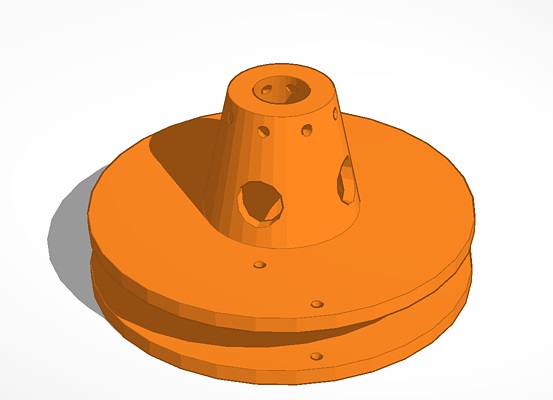

Supplement: Supplemental data [file Supp_Data.zip › Supplemental Information/3D Printer Parts/reel with holes.jpg]

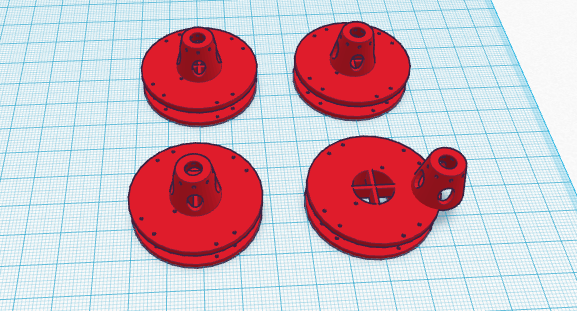

Supplement: Supplemental data [file Supp_Data.zip › Supplemental Information/3D Printer Parts/reel_magn_restrain.png]

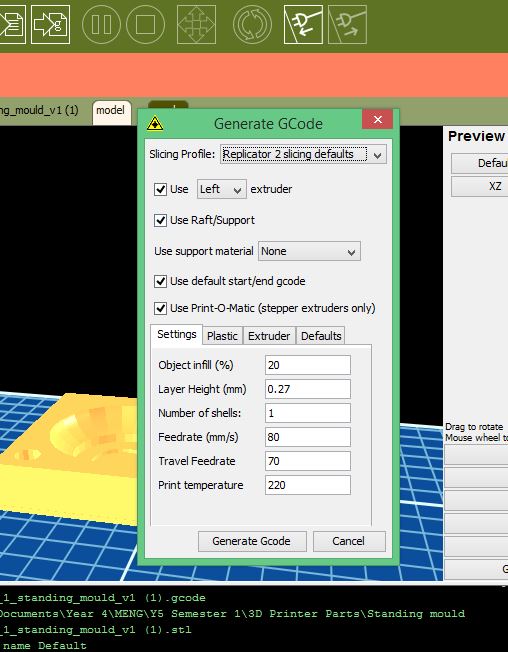

Supplement: Supplemental data [file Supp_Data.zip › Supplemental Information/3D Printer Parts/replicator g.JPG]

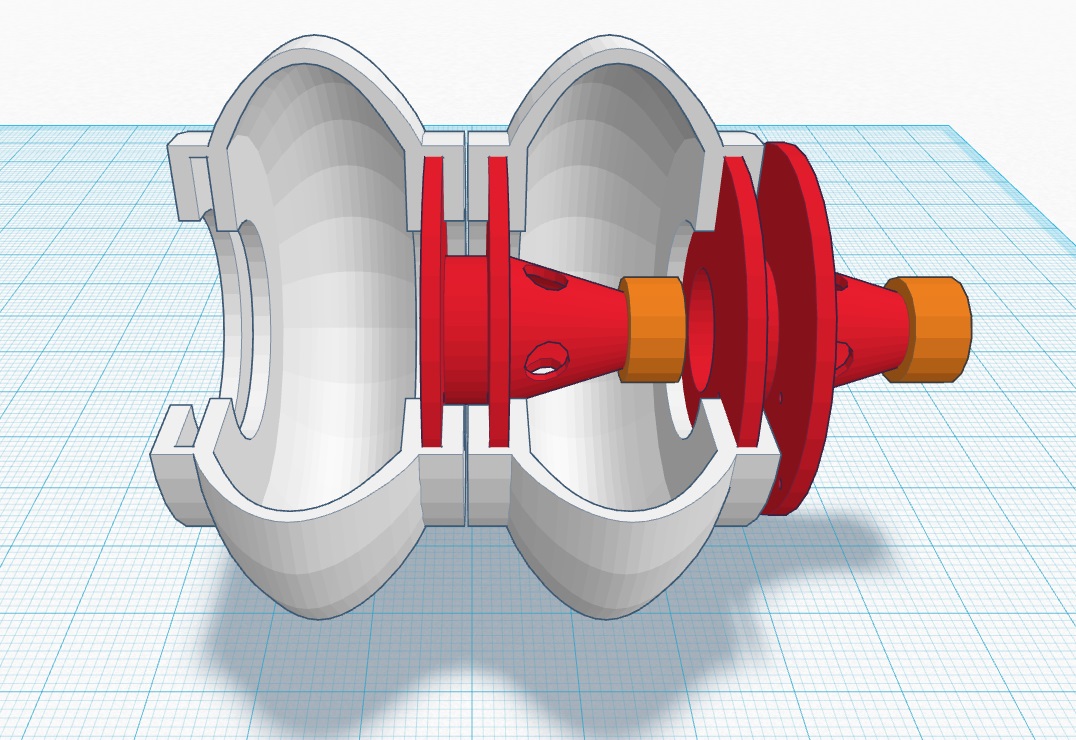

Supplement: Supplemental data [file Supp_Data.zip › Supplemental Information/3D Printer Parts/segment grip.jpg]

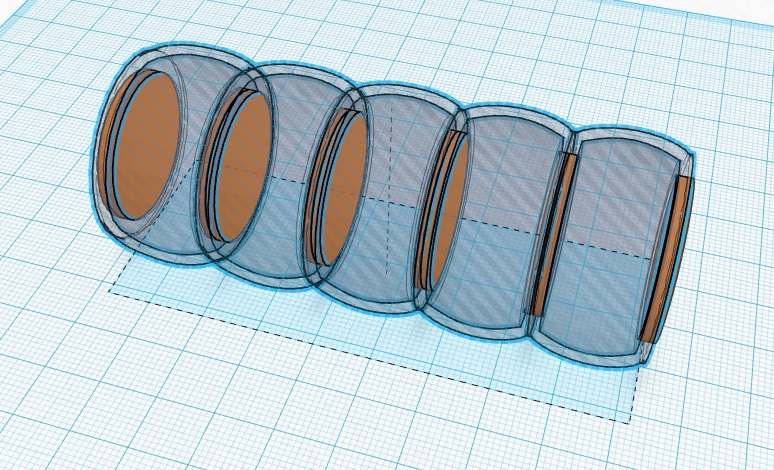

Supplement: Supplemental data [file Supp_Data.zip › Supplemental Information/3D Printer Parts/simple bot.jpg]

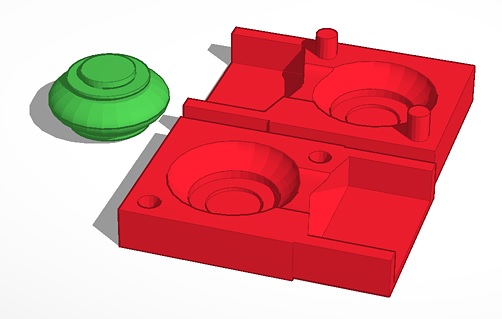

Supplement: Supplemental data [file Supp_Data.zip › Supplemental Information/3D Printer Parts/Standing mould 1/Untitled.jpg]

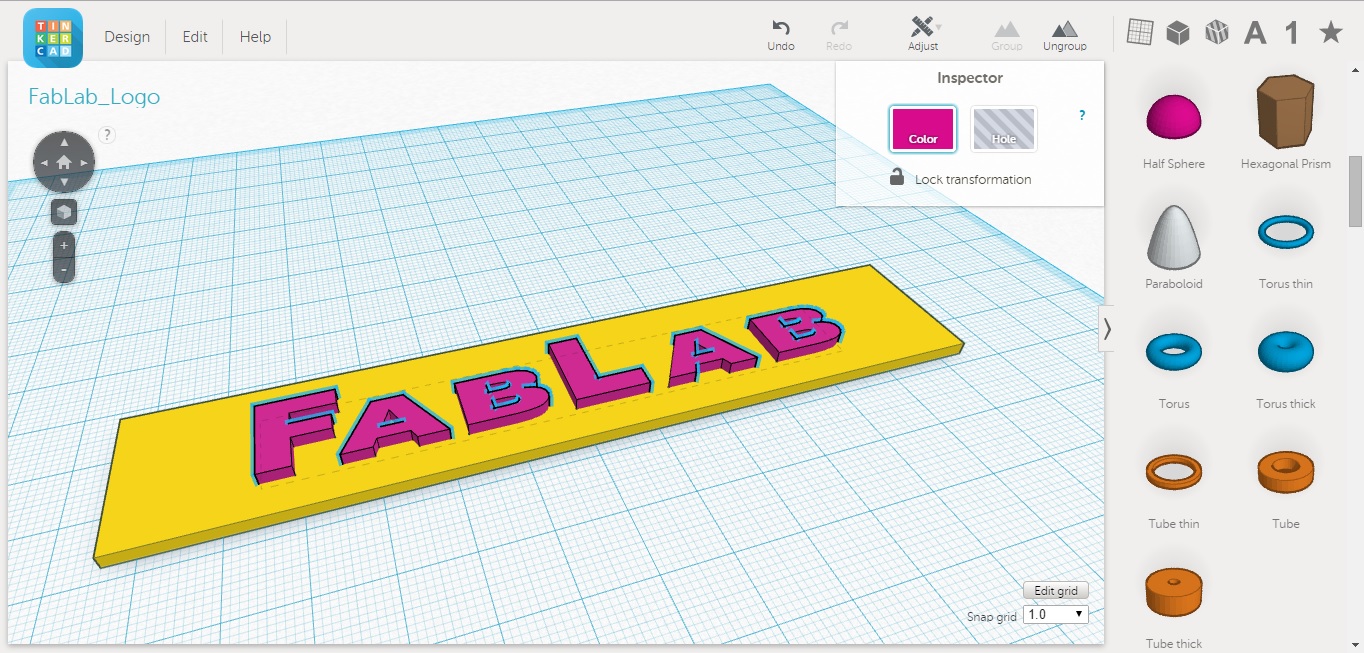

Supplement: Supplemental data [file Supp_Data.zip › Supplemental Information/3D Printer Parts/Stuff/fablab logo.jpg]

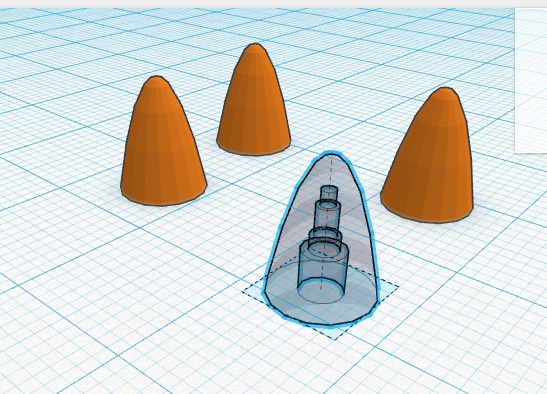

Supplement: Supplemental data [file Supp_Data.zip › Supplemental Information/3D Printer Parts/Stuff/pcb legs.jpg]

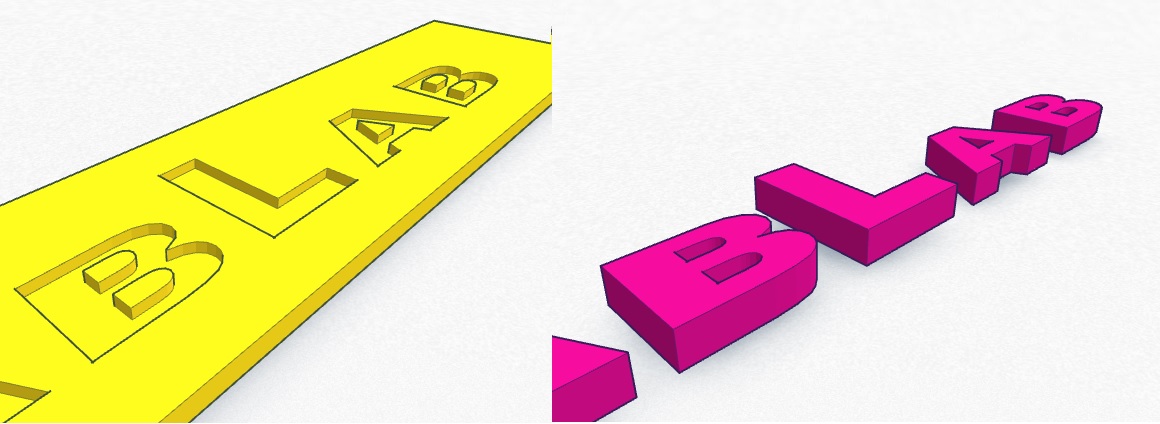

Supplement: Supplemental data [file Supp_Data.zip › Supplemental Information/3D Printer Parts/Stuff/Untitled.jpg]

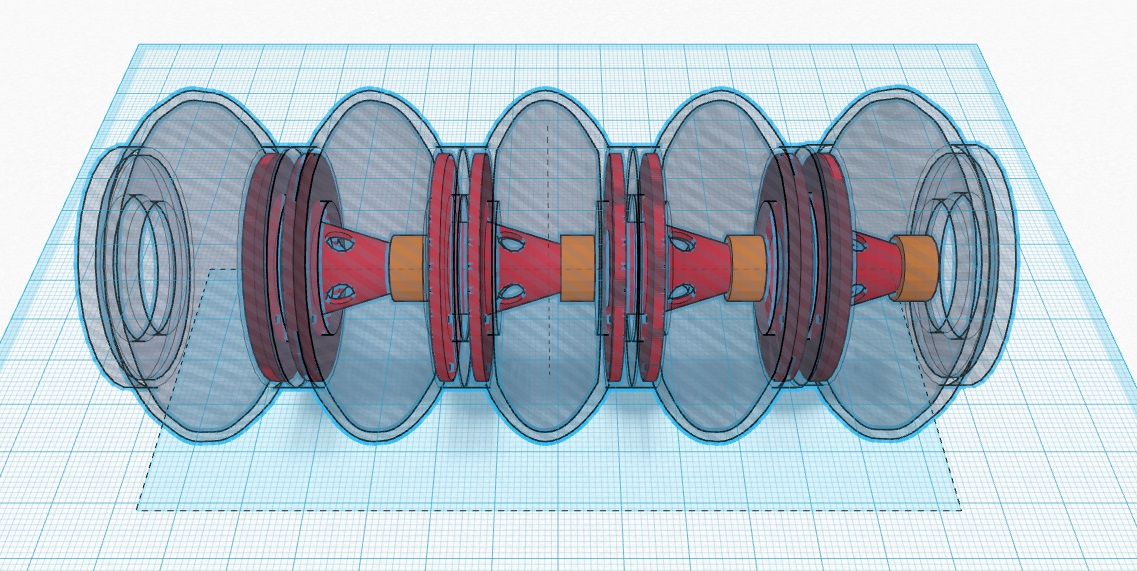

Supplement: Supplemental data [file Supp_Data.zip › Supplemental Information/3D Printer Parts/system 2 many.jpg]

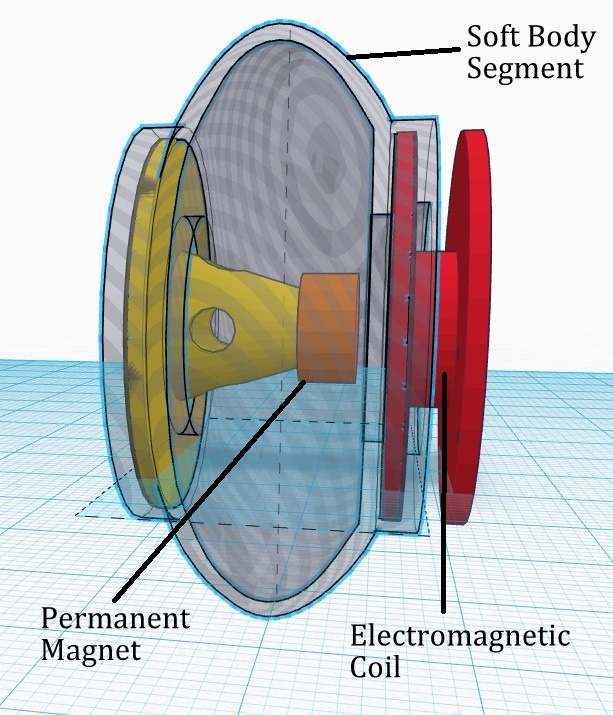

Supplement: Supplemental data [file Supp_Data.zip › Supplemental Information/3D Printer Parts/system 2.jpg]

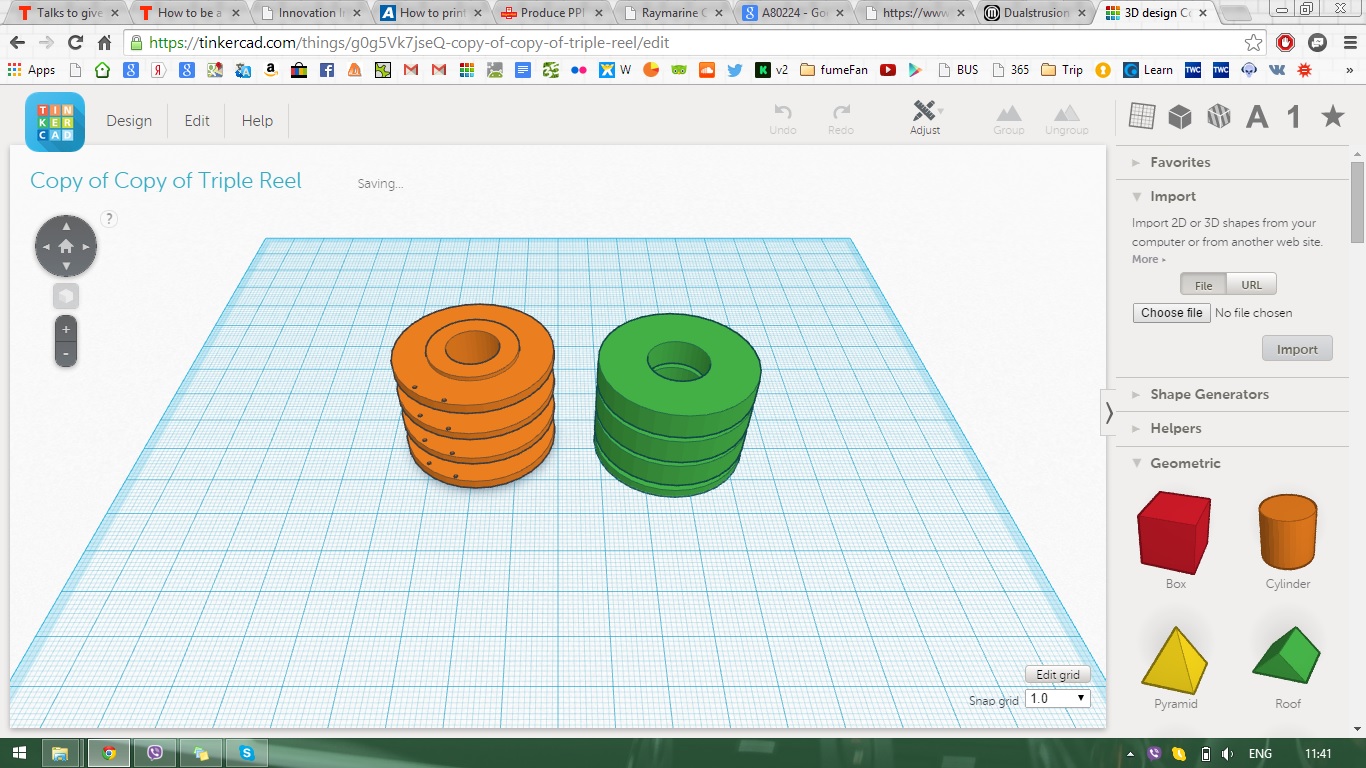

Supplement: Supplemental data [file Supp_Data.zip › Supplemental Information/3D Printer Parts/Tripple Reel/Bi Extruder/Untitled.jpg]

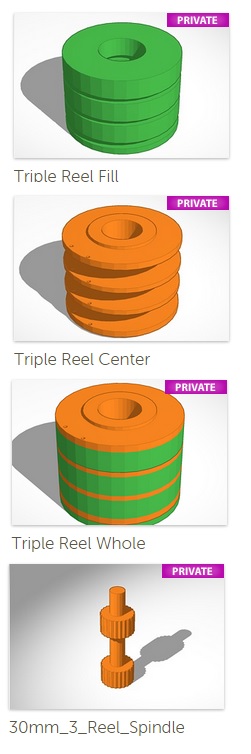

Supplement: Supplemental data [file Supp_Data.zip › Supplemental Information/3D Printer Parts/Tripple Reel/Bi Extruder/v2/Untitled.jpg]

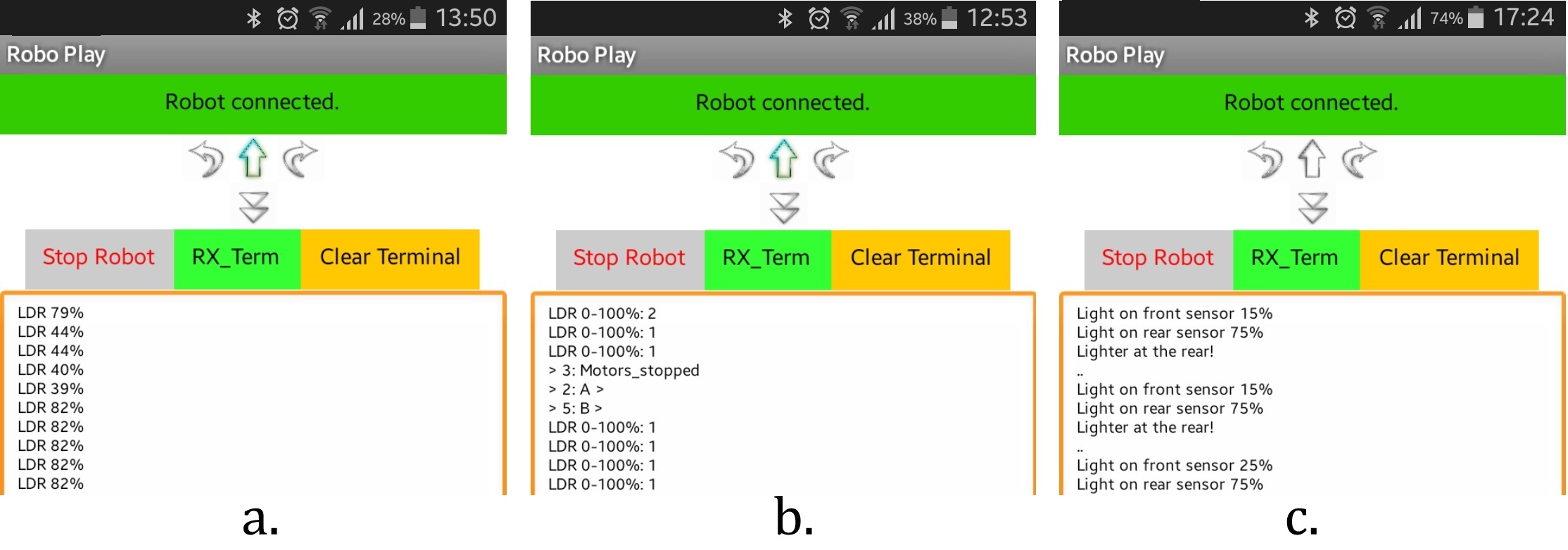

Supplement: Supplemental data [file Supp_Data.zip › Supplemental Information/Android App/3 screens.jpg]

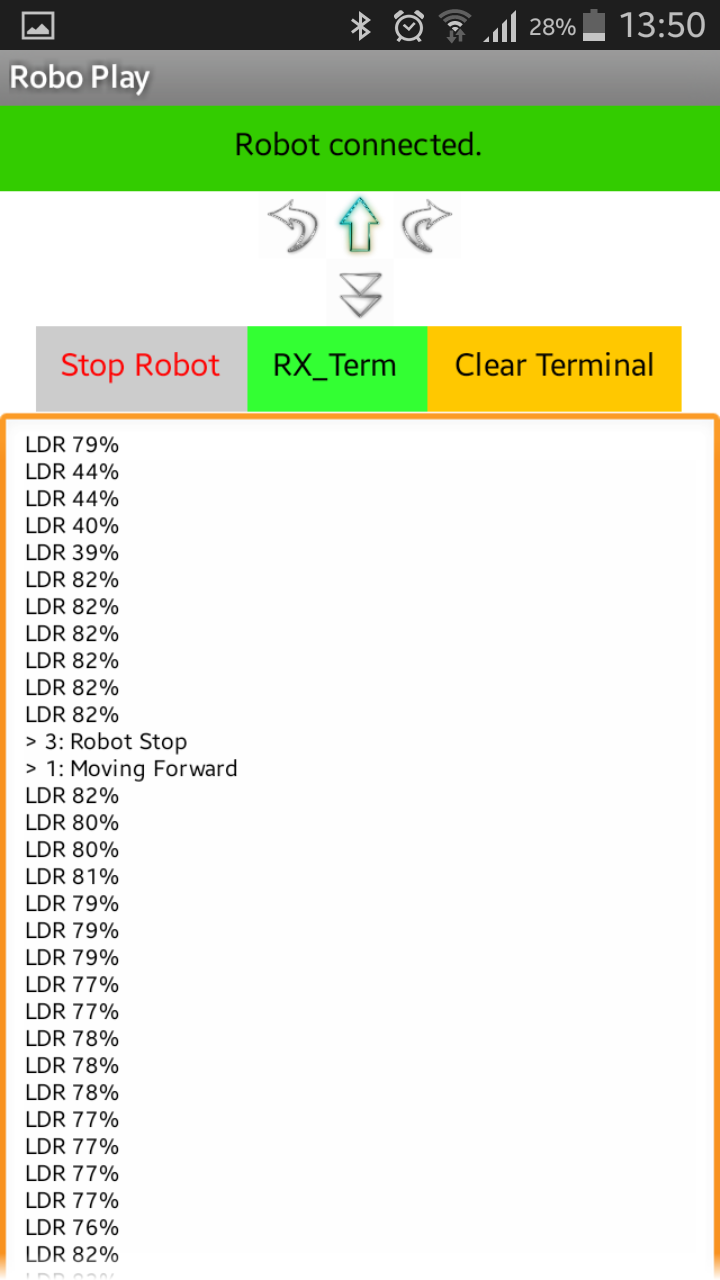

Supplement: Supplemental data [file Supp_Data.zip › Supplemental Information/Android App/Screenshot_2014-11-21-13-50-05.png]

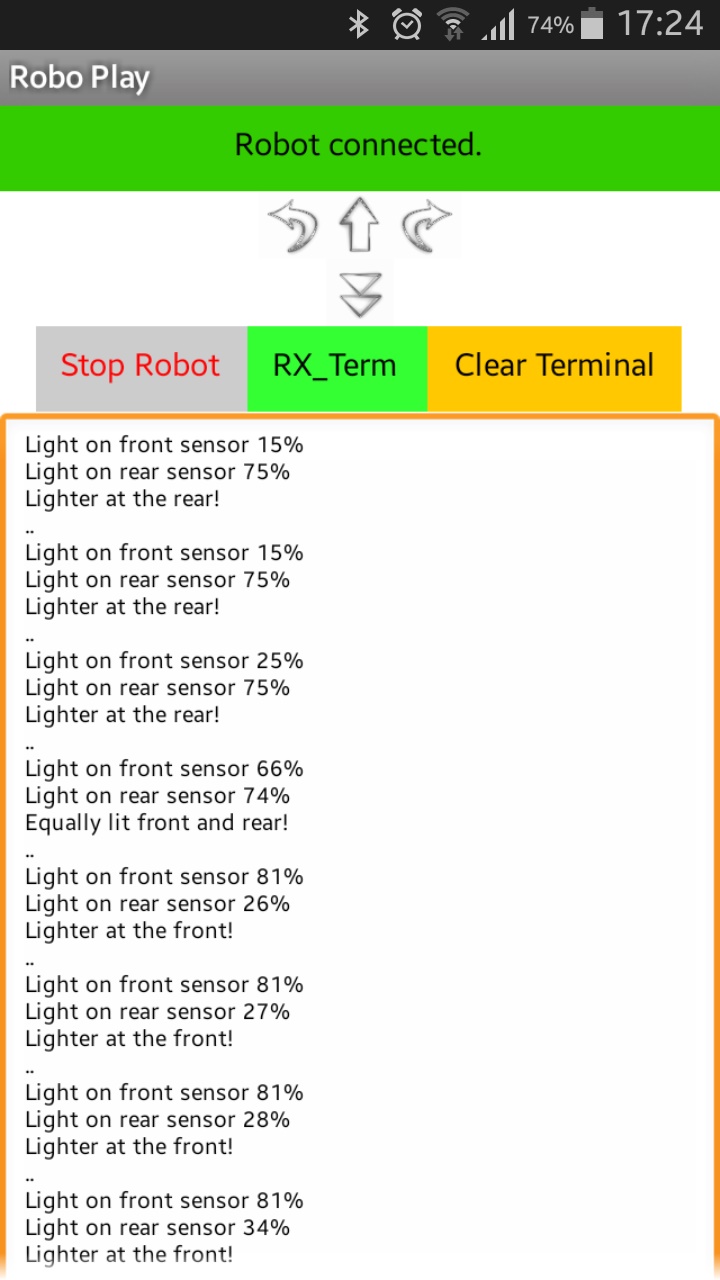

Supplement: Supplemental data [file Supp_Data.zip › Supplemental Information/Android App/Screenshot_2014-12-16-17-24-36.jpg]

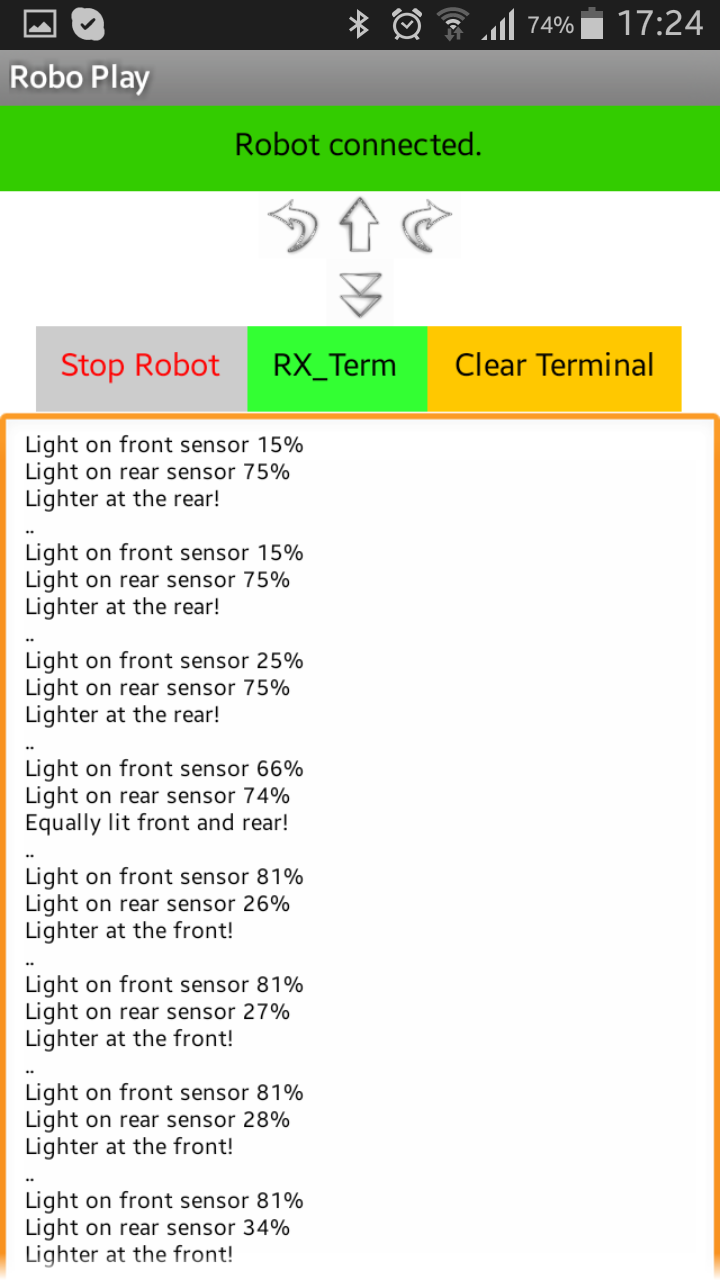

Supplement: Supplemental data [file Supp_Data.zip › Supplemental Information/Android App/Screenshot_2014-12-16-17-24-36.png]

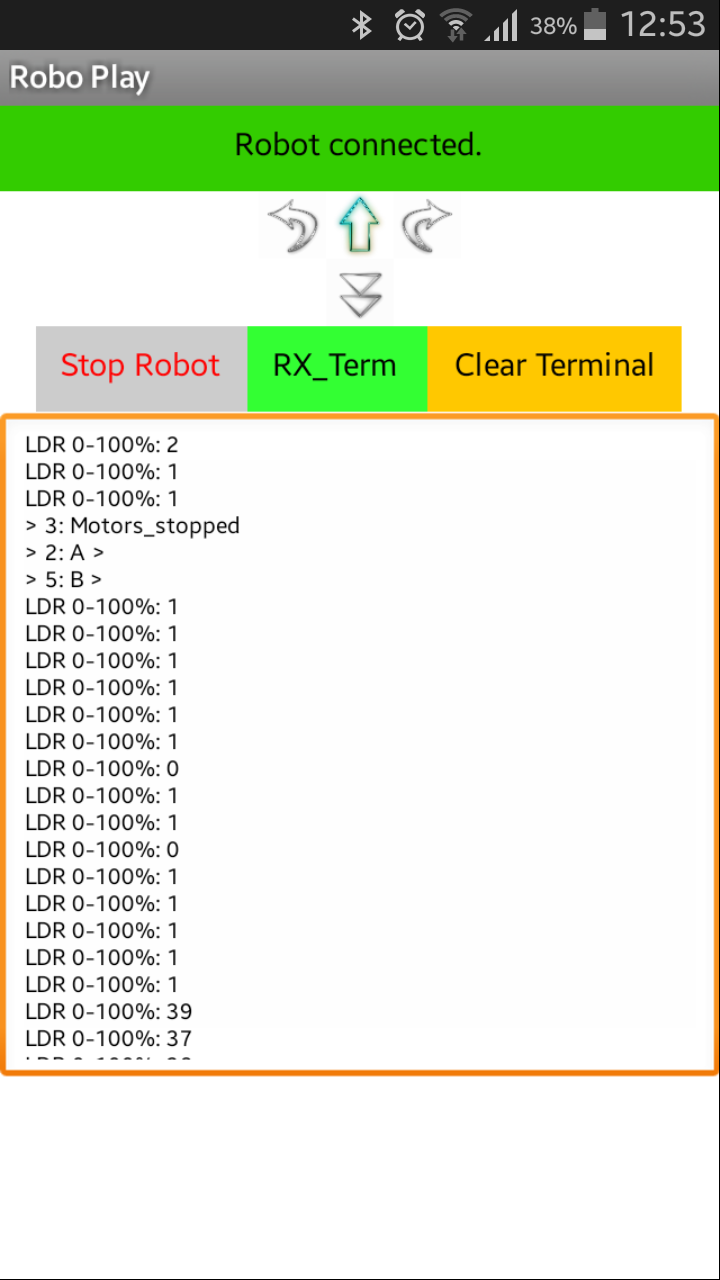

Supplement: Supplemental data [file Supp_Data.zip › Supplemental Information/Android App/Screenshot_2015-01-05-15-35-22.png]

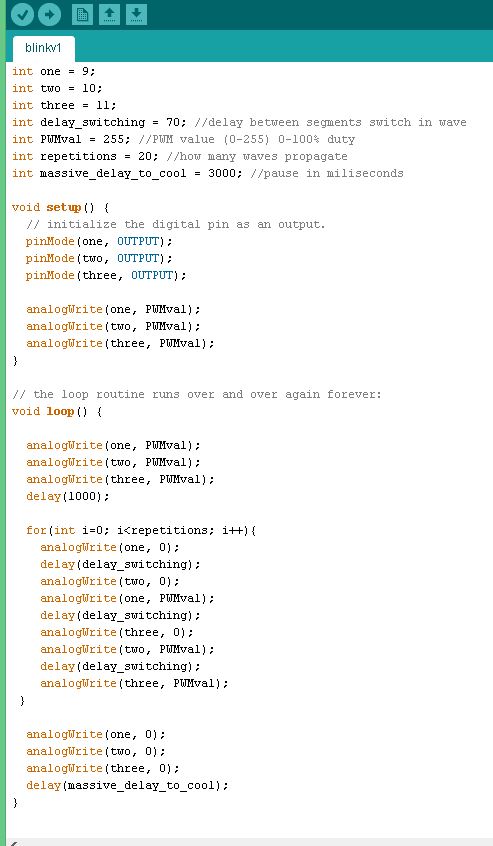

Supplement: Supplemental data [file Supp_Data.zip › Supplemental Information/Arduino Code/Bio-inspired Robot/3 120 deg phase/3 outputs single pole/Capture.JPG]

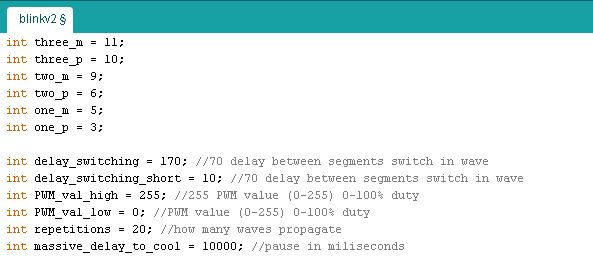

Supplement: Supplemental data [file Supp_Data.zip › Supplemental Information/Arduino Code/Bio-inspired Robot/3 120 deg phase/6 outputs 3 dualpolar/ddefinitions variables.JPG]

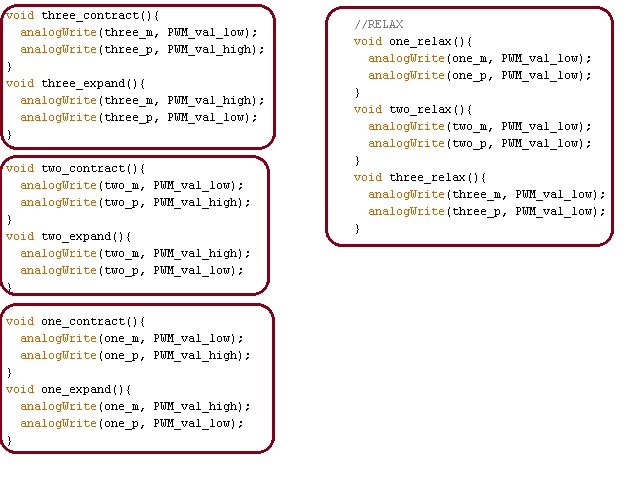

Supplement: Supplemental data [file Supp_Data.zip › Supplemental Information/Arduino Code/Bio-inspired Robot/3 120 deg phase/6 outputs 3 dualpolar/functions.jpg]

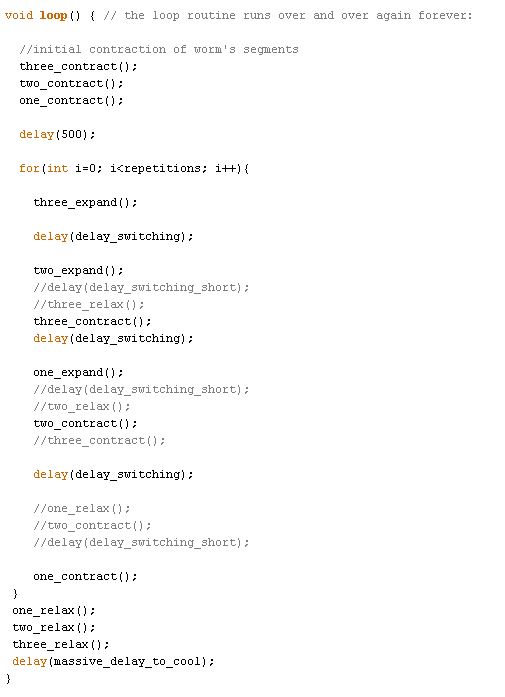

Supplement: Supplemental data [file Supp_Data.zip › Supplemental Information/Arduino Code/Bio-inspired Robot/3 120 deg phase/6 outputs 3 dualpolar/main loop.JPG]

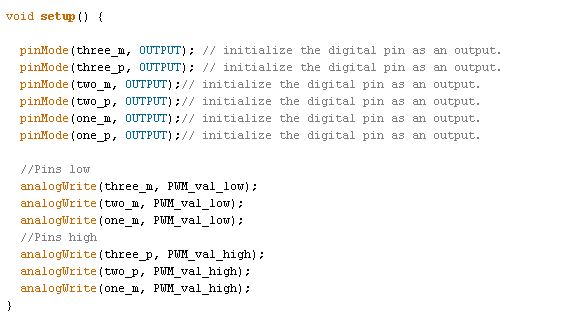

Supplement: Supplemental data [file Supp_Data.zip › Supplemental Information/Arduino Code/Bio-inspired Robot/3 120 deg phase/6 outputs 3 dualpolar/setup.JPG]

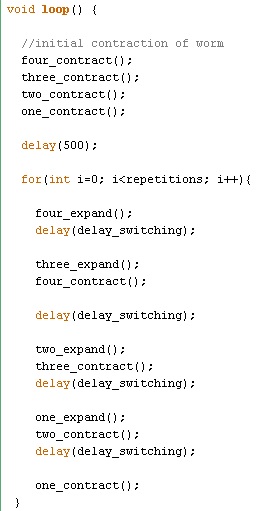

Supplement: Supplemental data [file Supp_Data.zip › Supplemental Information/Arduino Code/Bio-inspired Robot/3 120 deg phase/8 outputs 4 dualpolar/Untitled.jpg]

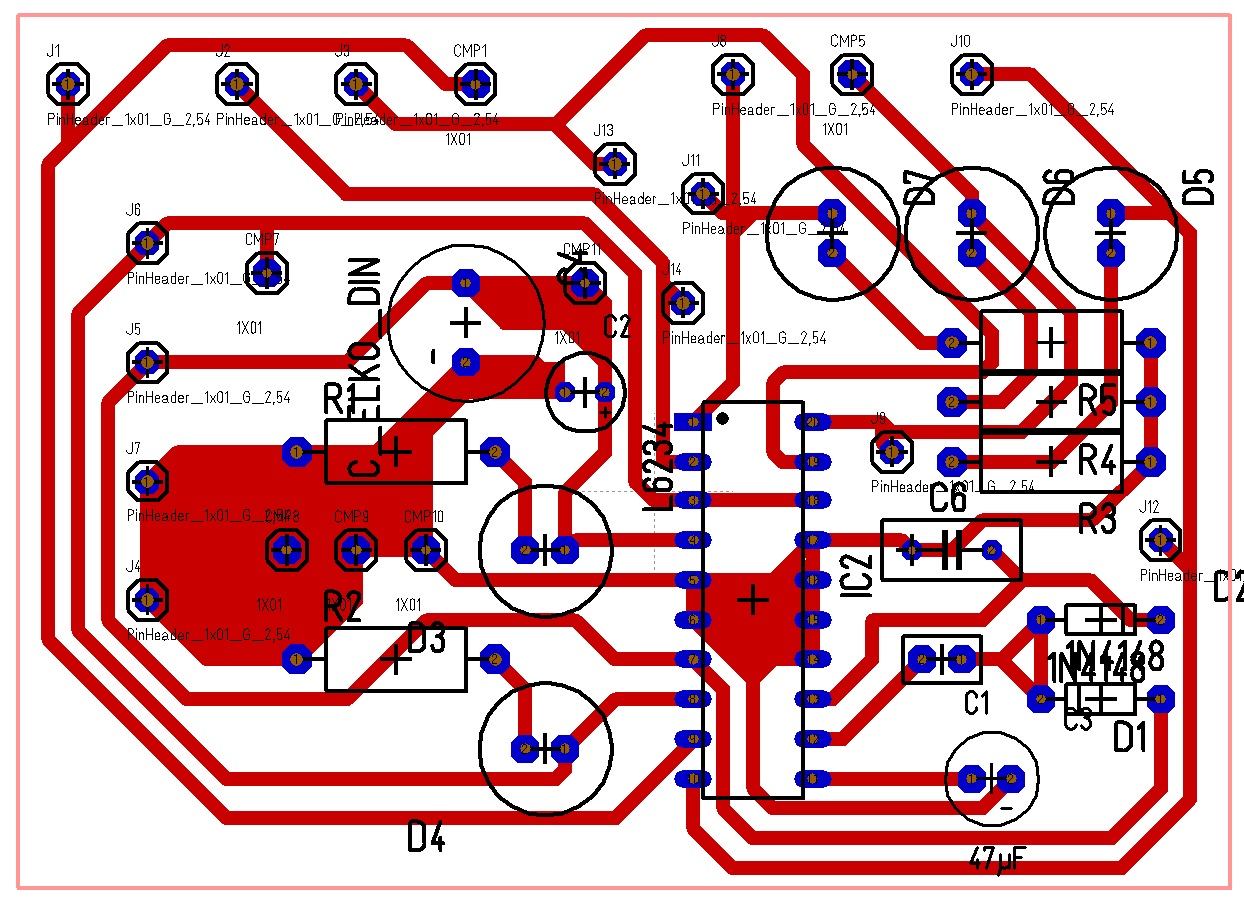

Supplement: Supplemental data [file Supp_Data.zip › Supplemental Information/PCB Schematics/1pcb drawing and leds.jpg]

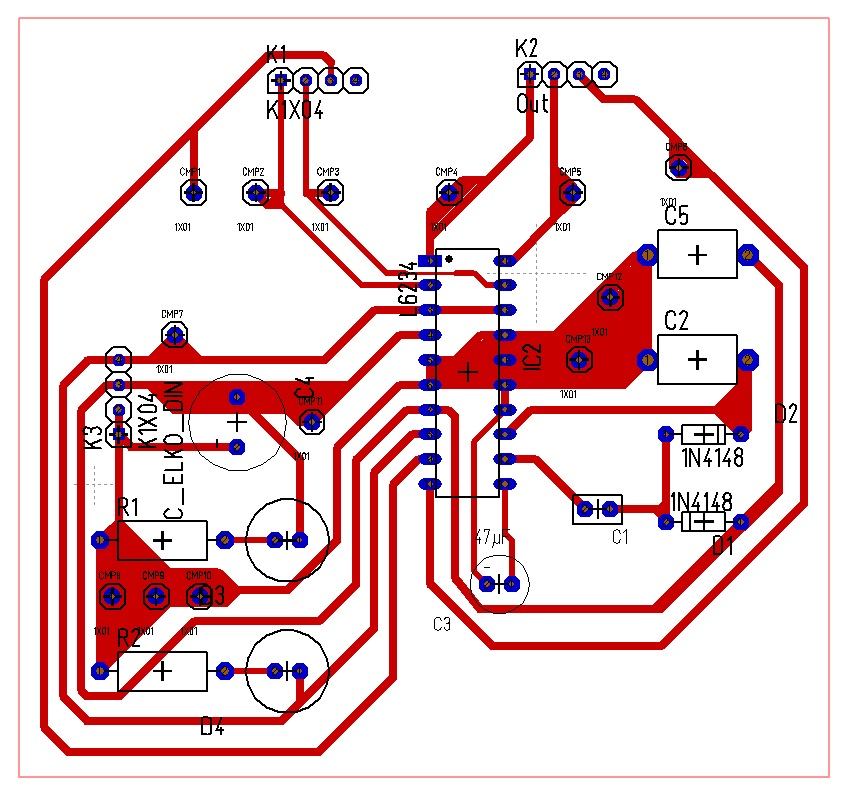

Supplement: Supplemental data [file Supp_Data.zip › Supplemental Information/PCB Schematics/1pcb drawing.jpg]

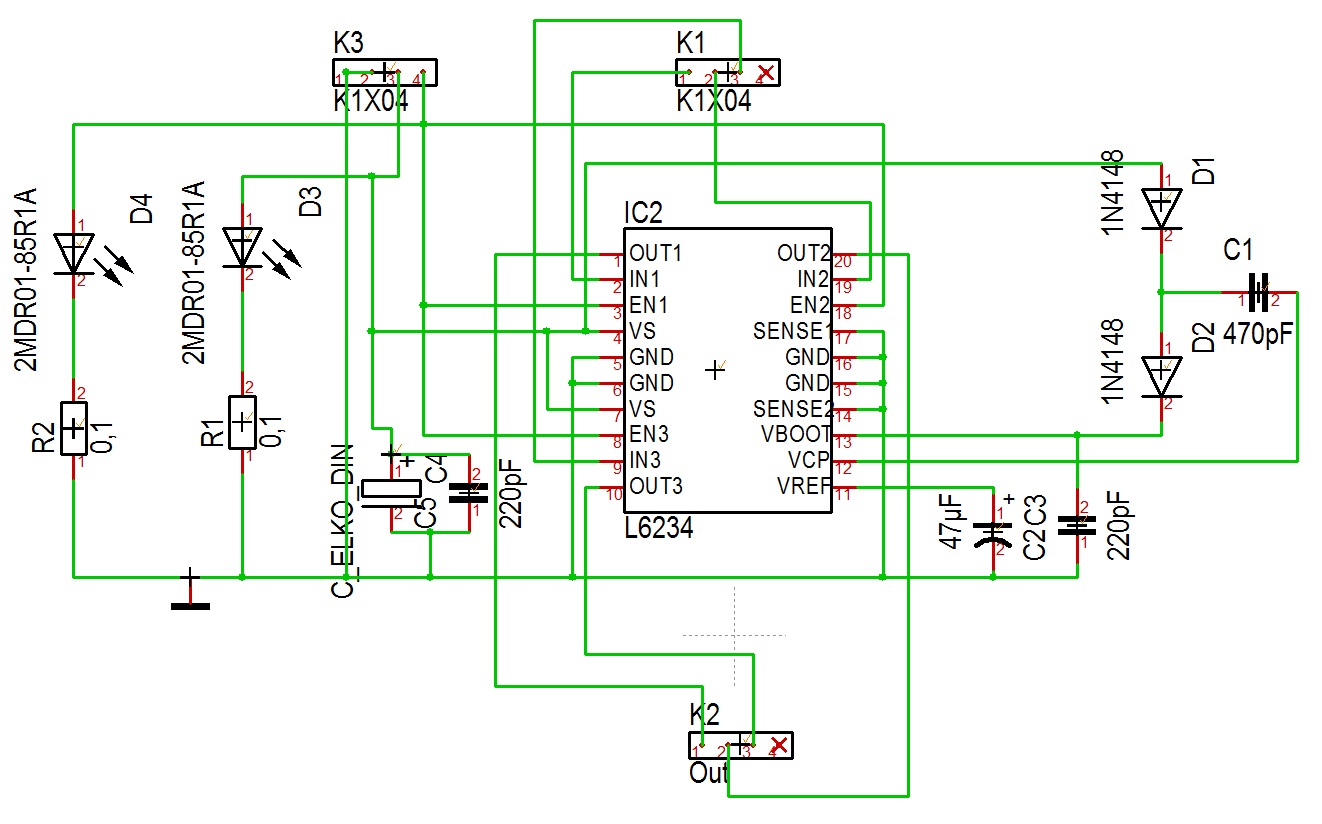

Supplement: Supplemental data [file Supp_Data.zip › Supplemental Information/PCB Schematics/1pcb.jpg]

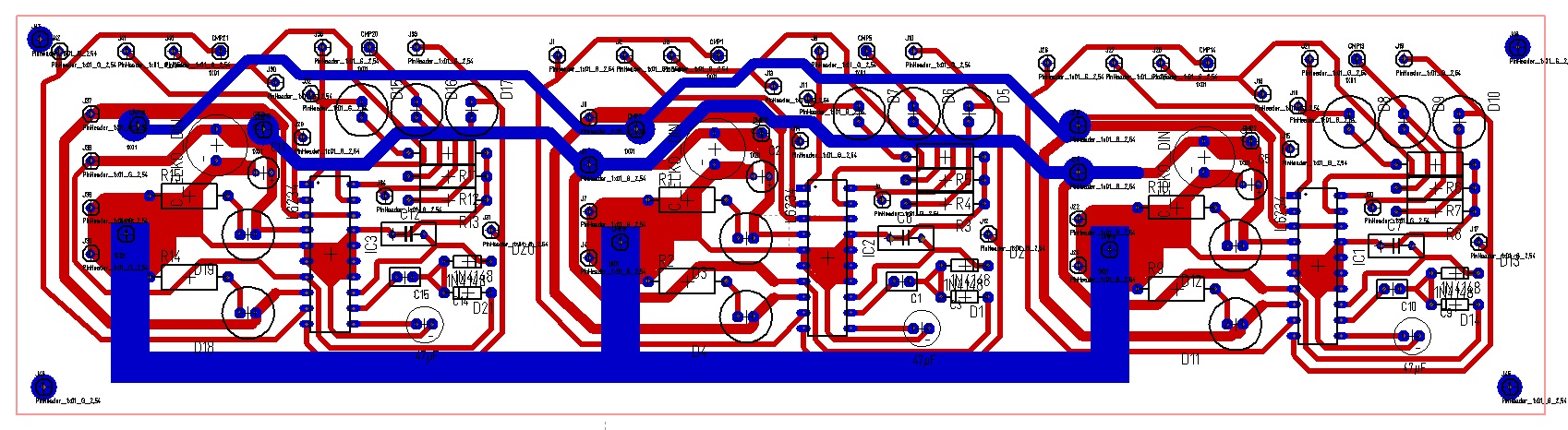

Supplement: Supplemental data [file Supp_Data.zip › Supplemental Information/PCB Schematics/3pcb drawing 1st version.jpg]

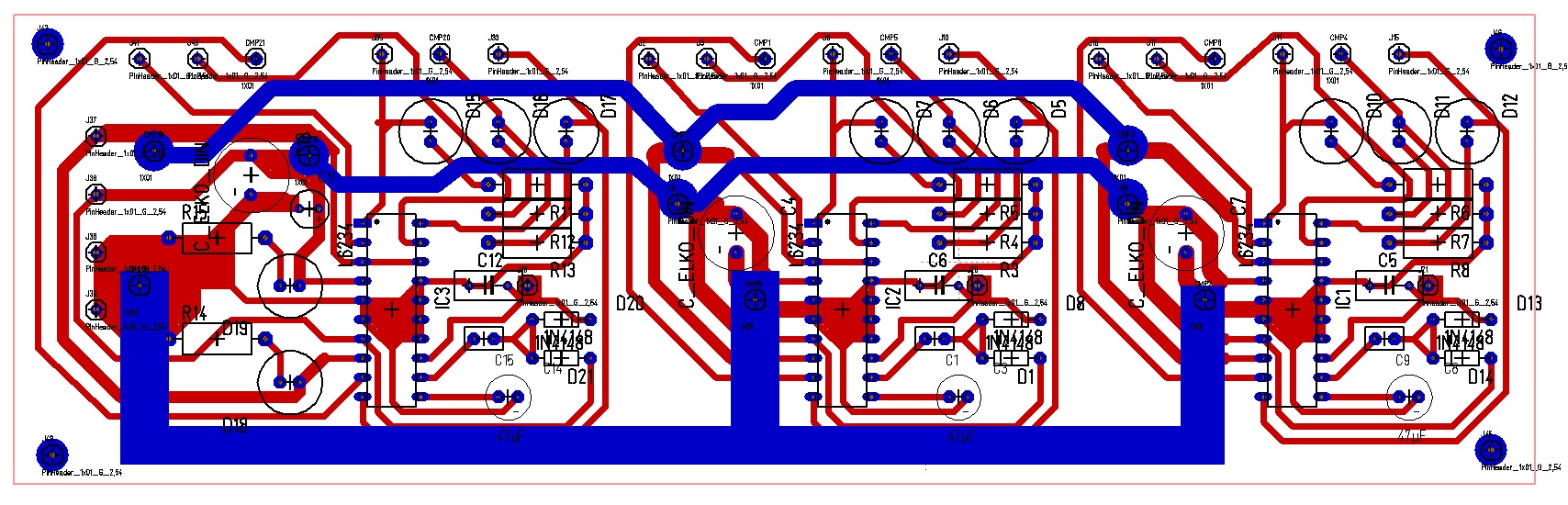

Supplement: Supplemental data [file Supp_Data.zip › Supplemental Information/PCB Schematics/3pcb drawing.jpg]

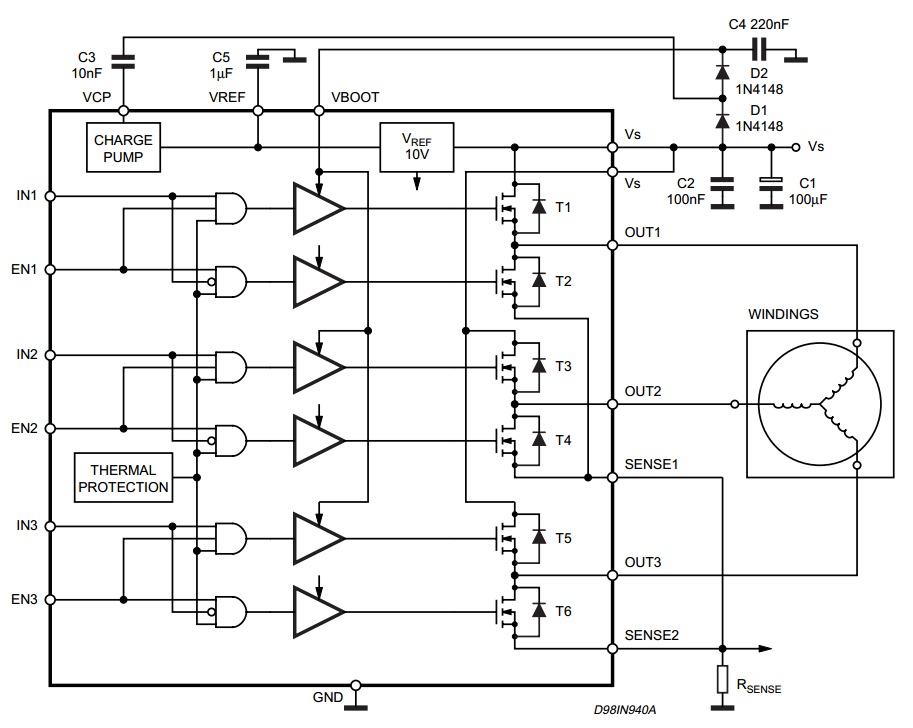

Supplement: Supplemental data [file Supp_Data.zip › Supplemental Information/PCB Schematics/driver.jpg]

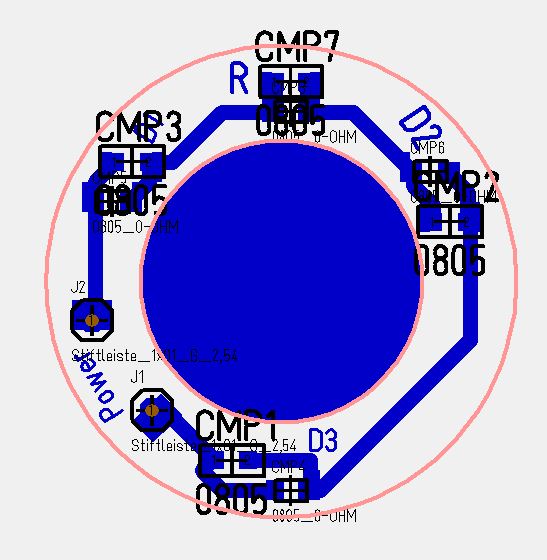

Supplement: Supplemental data [file Supp_Data.zip › Supplemental Information/PCB Schematics/LED circle PCB/Capture.JPG]

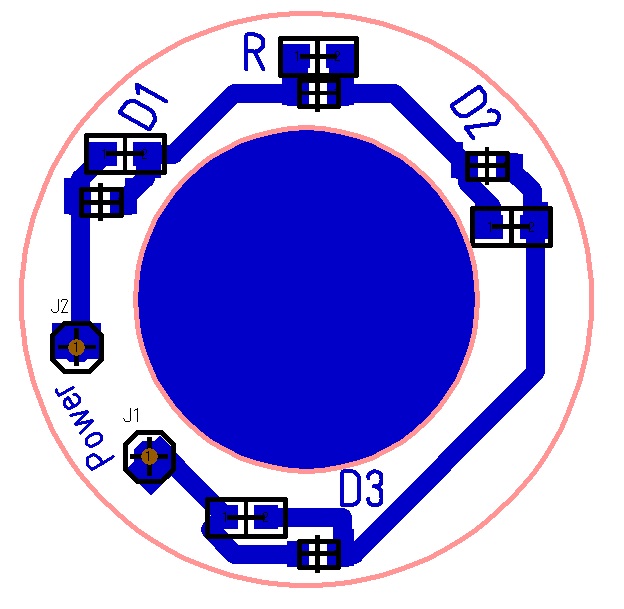

Supplement: Supplemental data [file Supp_Data.zip › Supplemental Information/PCB Schematics/LED circle PCB/led pcb.jpg]

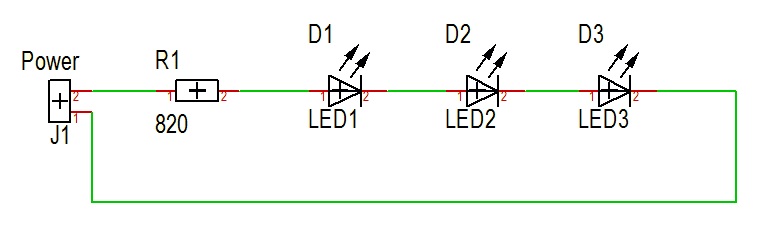

Supplement: Supplemental data [file Supp_Data.zip › Supplemental Information/PCB Schematics/LED circle PCB/led schematic.jpg]

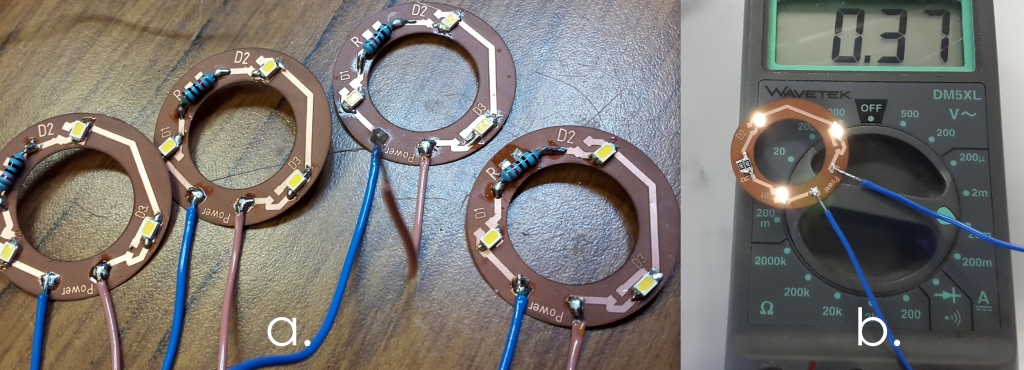

Supplement: Supplemental data [file Supp_Data.zip › Supplemental Information/PCB Schematics/LED circle PCB/Y5 Semester 11-001.jpg]

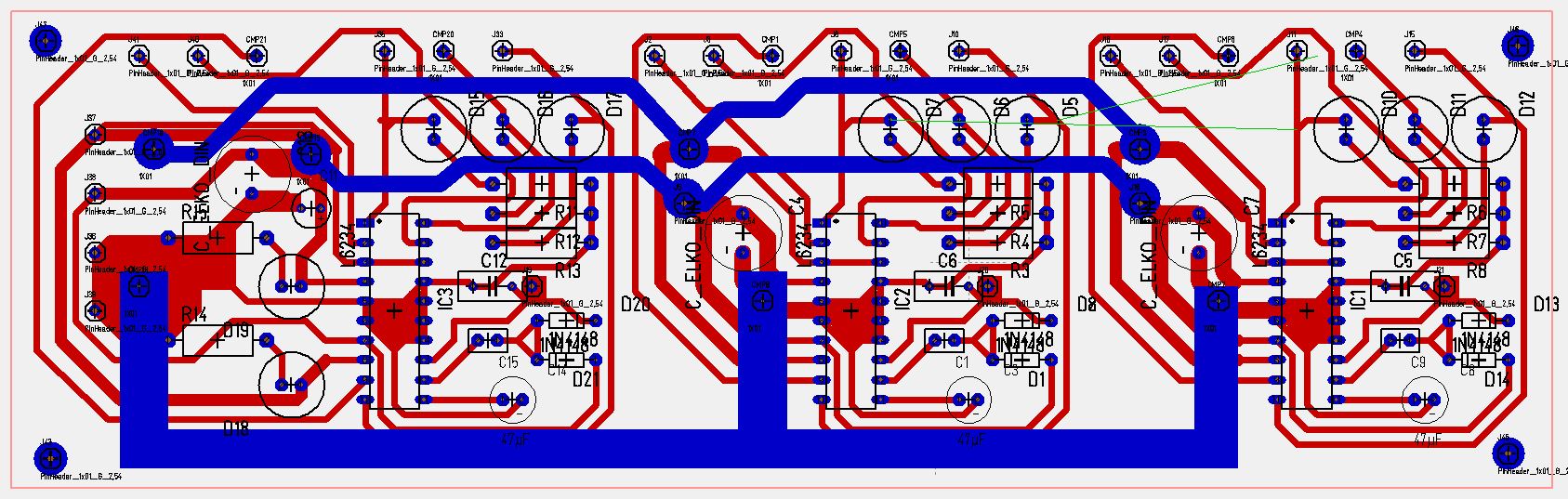

Supplement: Supplemental data [file Supp_Data.zip › Supplemental Information/PCB Schematics/Tripple Driver/Capture.JPG]

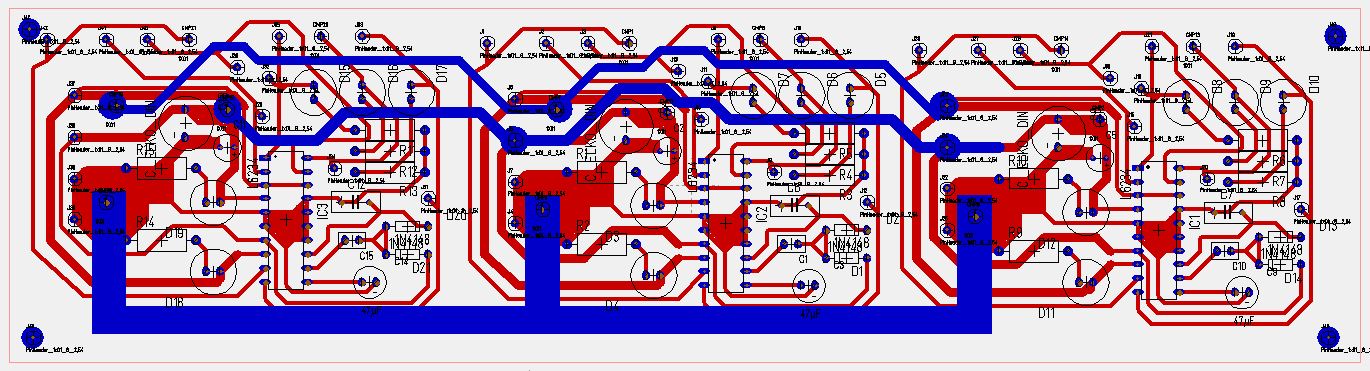

Supplement: Supplemental data [file Supp_Data.zip › Supplemental Information/PCB Schematics/Tripple Driver/pcb 3ple.JPG]

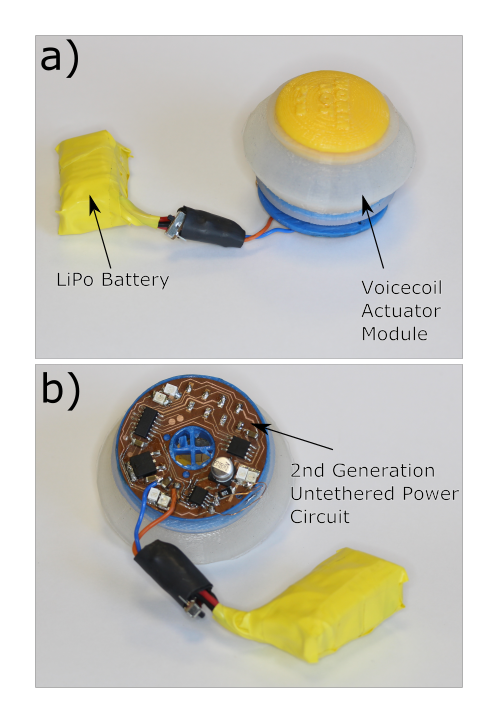

Supplement: Supplemental data [file Supp_Fig7.tif]

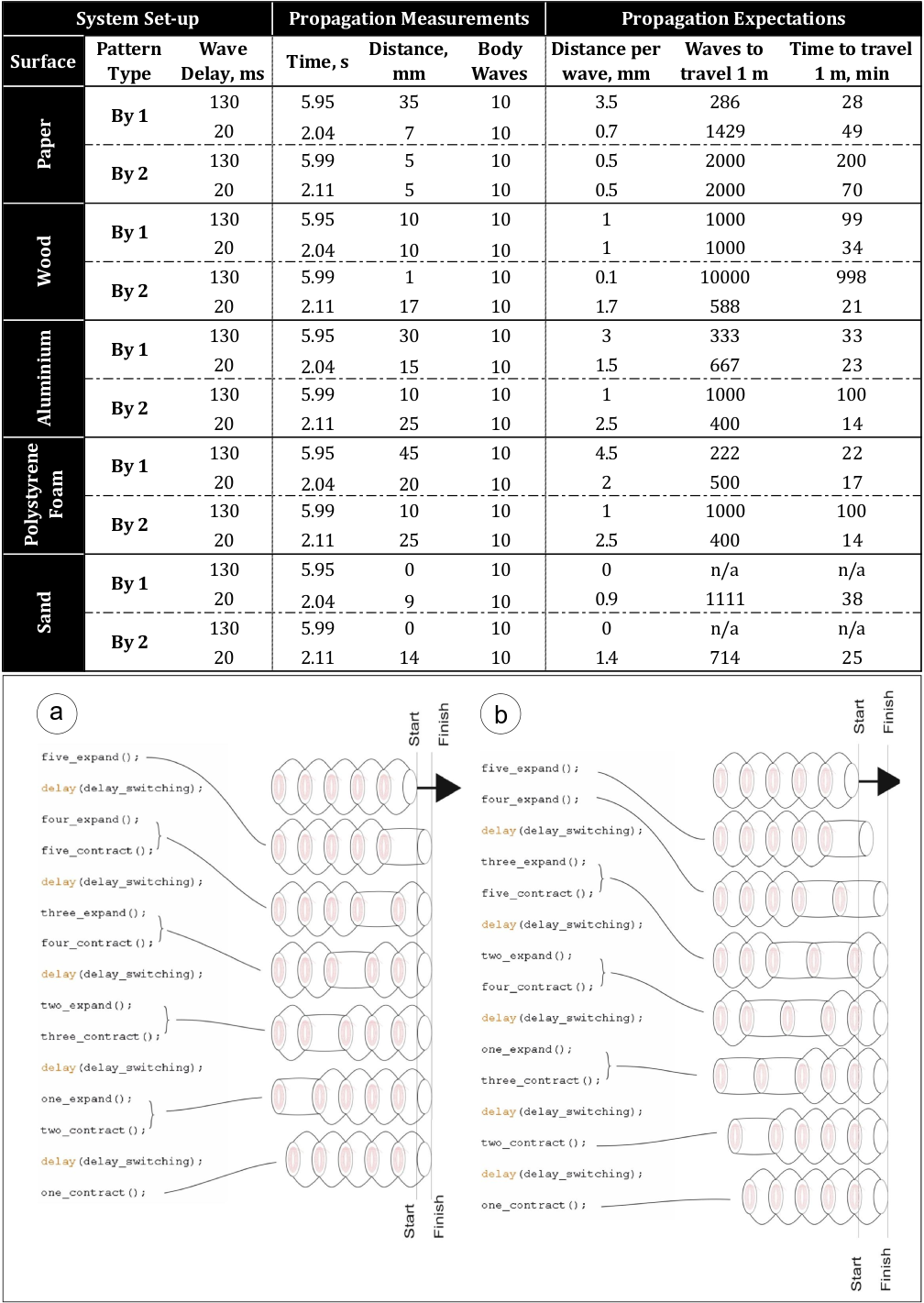

Supplement: Supplemental data [file Supp_Fig4.tif]

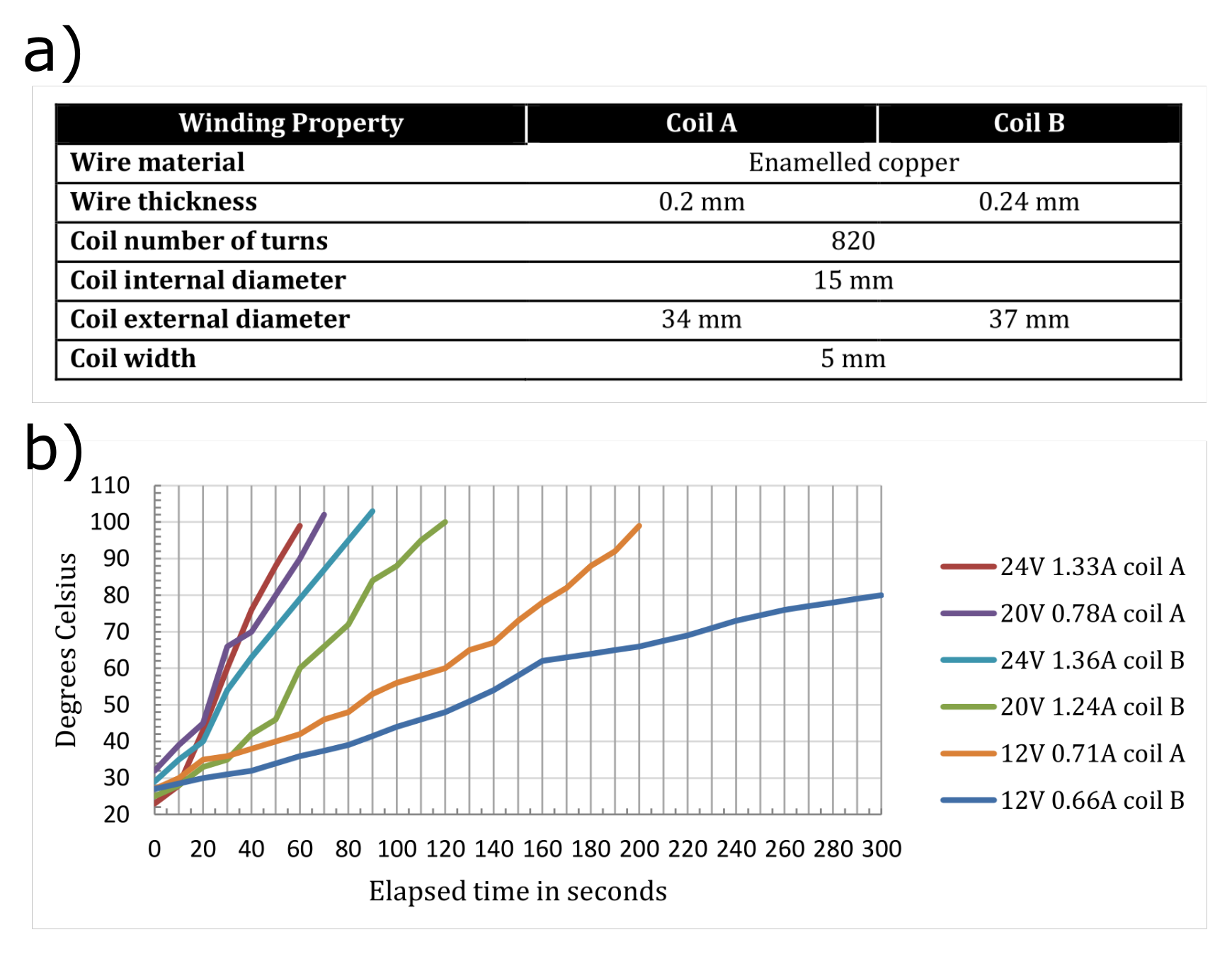

Supplement: Supplemental data [file Supp_Fig6.tif]

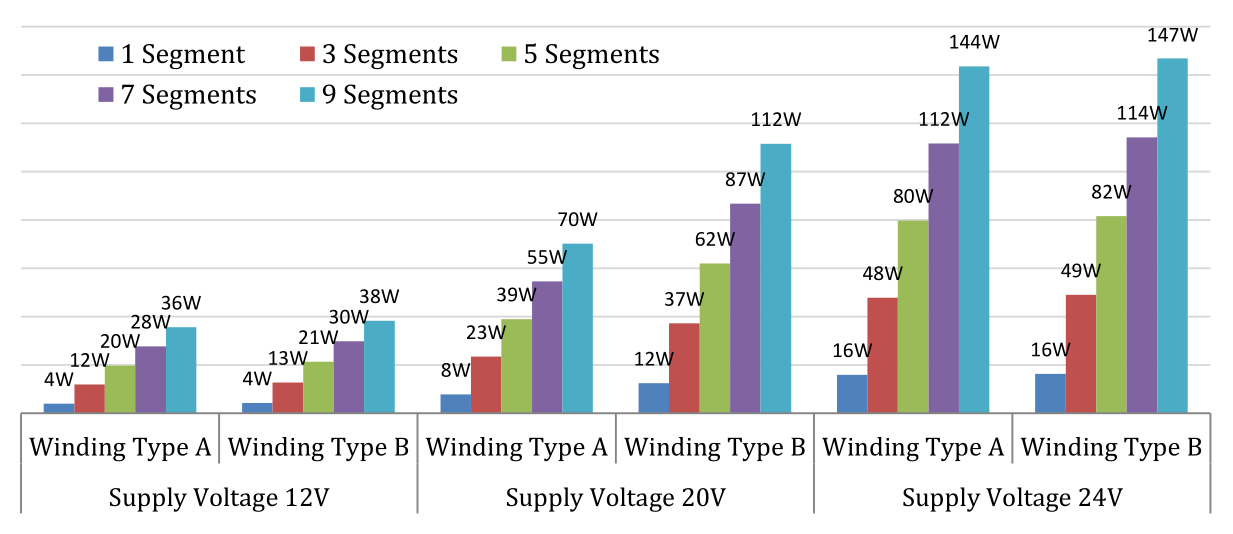

Supplement: Supplemental data [file Supp_Fig8.tif]

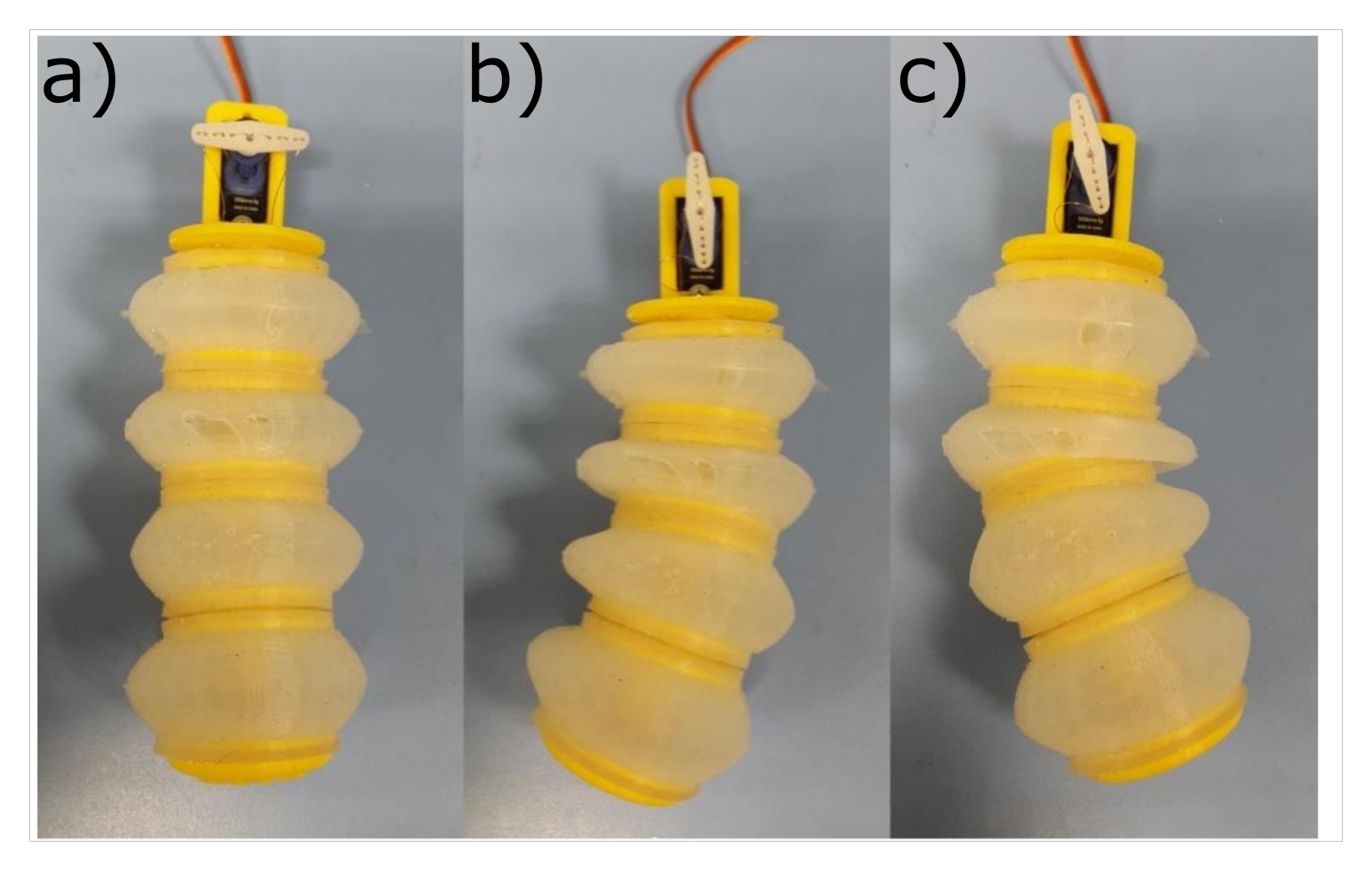

Supplement: Supplemental data [file Supp_Fig9.tif]

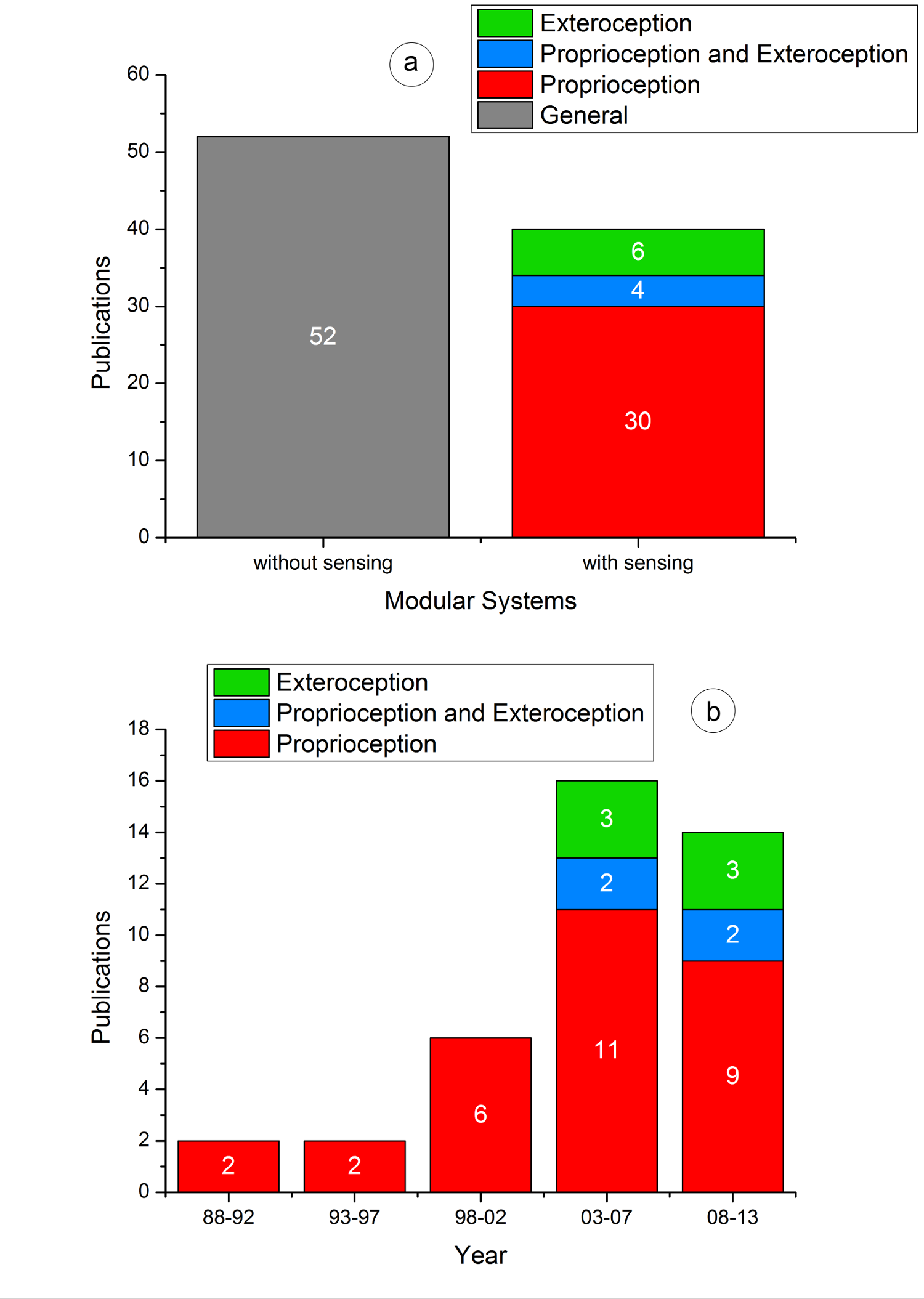

Supplement: Supplemental data [file Supp_Fig3.tif]

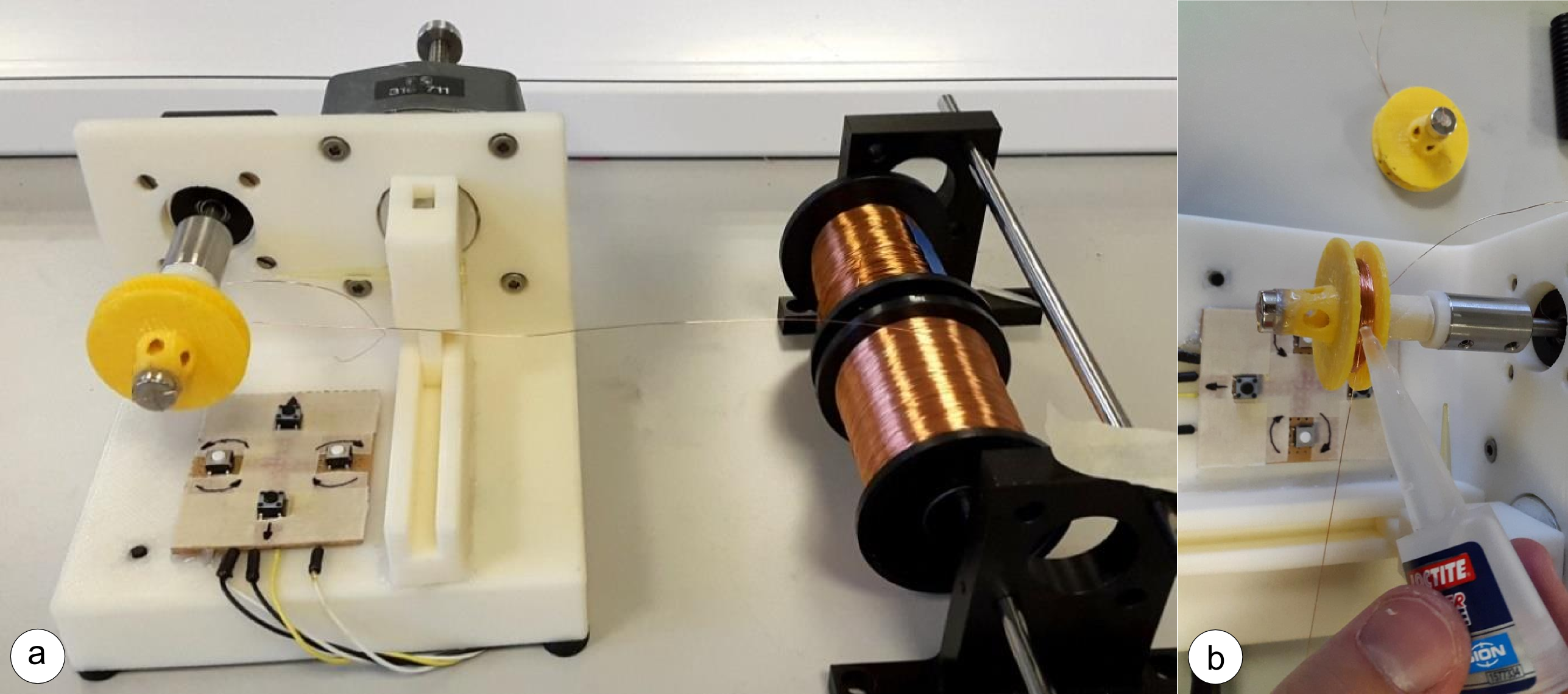

Supplement: Supplemental data [file Supp_Fig1.tif]

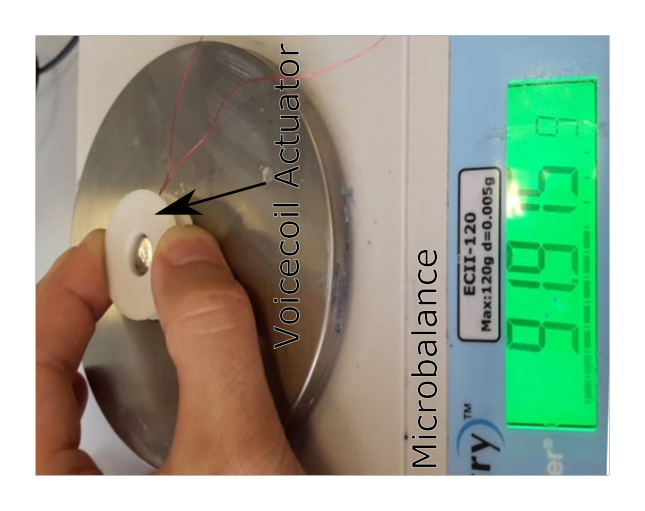

Supplement: Supplemental data [file Supp_Fig5.tif]

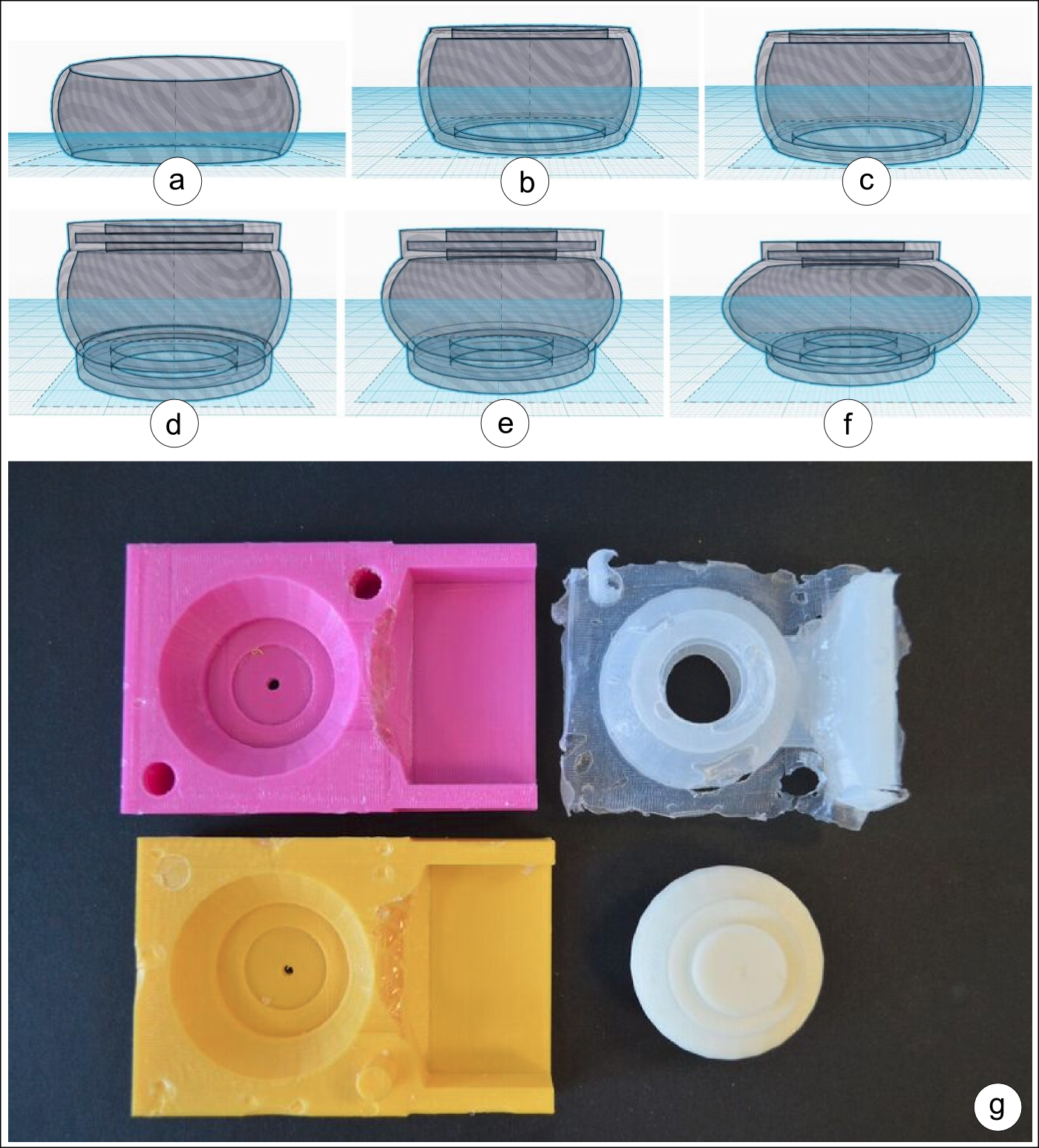

Supplement: Supplemental data [file Supp_Fig2.tif]
